# Exploratory study on the relationship between urinary sodium/potassium ratio, salt intake, and the antihypertensive effect of esaxerenone: the ENaK Study

**DOI:** 10.1038/s41440-023-01519-0

**Published:** 2024-01-11

**Authors:** Tomohiro Katsuya, Yoshito Inobe, Kazuaki Uchiyama, Tetsuo Nishikawa, Kunio Hirano, Mitsutoshi Kato, Toshiki Fukui, Tsuguru Hatta, Arata Iwasaki, Hajime Ishii, Toshiyuki Sugiura, Takashi Taguchi, Ayumi Tanabe, Kotaro Sugimoto, Tatsuo Shimosawa, on behalf of the ENaK investigators

**Affiliations:** 1 Katsuya Clinic, Amagasaki, Japan; 2 Inobe Funai Clinic, Oita, Japan; 3 Uchiyama Clinic, Joetsu, Japan; 4 Nishikawa Clinic, Yokohama, Japan; 5 Hirano Clinic, Morioka, Japan; 6 Kato Clinic of Internal Medicine, Tokyo, Japan; 7 Olive Takamatsu Medical Clinic, Takamatsu, Japan; 8 Hatta Medical Clinic, Kyoto, Japan; 9 Asamoto Internal Medicine Clinic, Kyoto, Japan; 10 Kashinoki Internal Medicine, Date, Japan; 11 Medical Corporation Association Koukeikai Sugiura Clinic, Kawaguchi, Japan; 12 Daiichi Sankyo Co., Ltd, Tokyo, Japan; 13 Department of Clinical Laboratory, School of Medicine, International University of Health and Welfare, Narita, Japan

**Keywords:** Esaxerenone, Estimated 24-h urinary sodium excretion, Hypertension, Mineralocorticoid receptor blocker, Urinary sodium/potassium ratio

## Abstract

Excessive salt intake is one of the causes of hypertension, and reducing salt intake is important for managing the risk of hypertension and subsequent cardiovascular events. Esaxerenone, a mineralocorticoid receptor blocker, has the potential to exert an antihypertensive effect in hypertensive patients with excessive salt intake, but evidence is still lacking, especially in clinical settings. We aimed to determine if baseline sodium/potassium ratio and baseline estimated 24-h urinary sodium excretion can predict the antihypertensive effect of esaxerenone in patients with essential hypertension inadequately controlled with an angiotensin receptor blocker (ARB) or a calcium channel blocker (CCB). This was an exploratory, open-label, interventional study with a 4-week observation period and a 12-week treatment period. Esaxerenone was orally administered once daily in accordance with the Japanese package insert. In total, 126 patients met the eligibility criteria and were enrolled (ARB subcohort, 67; CCB subcohort, 59); all were included in the full analysis set (FAS) and safety analysis. In the FAS, morning home systolic blood pressure (SBP)/diastolic blood pressure (DBP) significantly decreased from baseline to end of treatment (primary efficacy endpoint) (−11.9 ± 10.9/ − 6.4 ± 6.8 mmHg, both *p* < 0.001); a similar trend was observed in both subcohorts. Significant reductions were also shown in bedtime home and office SBP/DBP (all *p* < 0.001). Each BP change was consistent regardless of the urinary sodium/potassium ratio or estimated 24-h urinary sodium excretion at baseline. The urinary albumin-creatinine ratio (UACR) and N-terminal pro-brain natriuretic peptide (NT-proBNP) significantly decreased from baseline to Week 12 in the total population and both subcohorts. No new safety concerns were raised. Esaxerenone significantly decreased morning home, bedtime home, and office BP; UACR; and NT-proBNP in this patient population, regardless of concomitant ARB or CCB use. The antihypertensive effect of esaxerenone was independent of the urinary sodium/potassium ratio and estimated 24-h urinary sodium excretion at baseline.

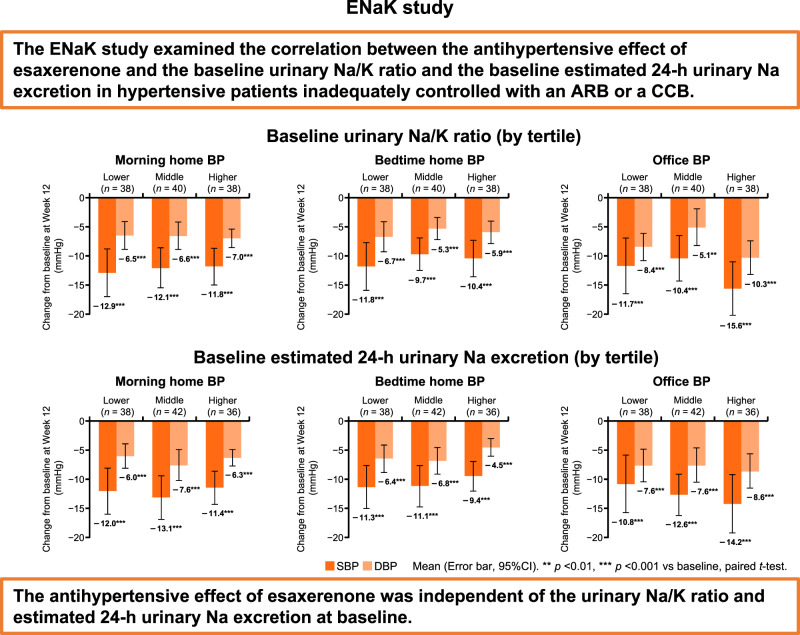

## Introduction

Excessive salt intake is one of the causes of hypertension and is associated with an increased risk of cardiovascular events [1, 2]. Given the generally higher salt intake and higher frequency of salt sensitivity due to genetic polymorphisms in Japanese populations vs Caucasians [3, 4], reducing salt intake, especially in Japanese individuals, is important to mitigate the risk of cardiovascular events and decrease blood pressure (BP) [2, 5–7]. Currently, urinary sodium (Na) excretion is used as an indirect indicator of salt intake [8]; however, it is difficult to easily identify patients with excessive salt intake in the daily clinical setting.

In Japan, angiotensin receptor blockers (ARBs) or calcium channel blockers (CCBs) are prescribed to hypertensive patients with no compelling indication [9], and many hypertensive patients with excessive salt intake are currently prescribed these drugs. However, the antihypertensive effect of ARBs is attenuated by excessive salt intake and may be inadequate [10–12]. Furthermore, current guidelines recommend the use of diuretics for treating patients with excessive salt intake [2], but diuretics are known to have metabolic side effects, especially at higher doses. Thus, there remains a need for better treatment options for hypertensive patients with excessive salt intake.

Excessive salt intake stimulates mineralocorticoid receptors (MRs) in the kidneys and causes salt-induced hypertension [13]. Studies in in vivo salt-loaded models have shown that activation of MRs via Rac1 as an aldosterone-independent pathway contributes to the development of hypertension and organ damage [14, 15]. Thus, MR blockade produces antihypertensive and organ-protective effects in salt-loaded animal models [16–19].

Clinically, MR blockers have also been shown to have strong antihypertensive effects in patients with excessive salt intake or low-renin hypertension [20, 21], and urinary Na excretion has been reported to positively correlate with the antihypertensive effect of the MR blocker spironolactone and eplerenone [20, 22]. Based on these findings, MR blockers are expected to be a good antihypertensive treatment option for patients with excessive salt intake. However, there is no known method to diagnose MR-activated patients in a clinical setting, just as there is no suitable approach to identify patients with excessive salt intake. To address this situation, the urinary sodium/potassium ratio (Na/K ratio) may be used as an indirect indicator of MR activation because MR promotes Na reabsorption and K excretion through aldosterone binding in the renal tubules [23, 24].

Esaxerenone is a nonsteroidal MR blocker that has shown BP-lowering activity in hypertensive patients with a wide variety of characteristics [25–32], and renoprotective effects in hypertensive patients with type 2 diabetes mellitus and albuminuria [29, 31, 32]. In a *post hoc* analysis of a long-term phase 3 study of esaxerenone [33], esaxerenone monotherapy showed a sustained and stable antihypertensive effect by improving salt and water retention in hypertensive patients with higher baseline Na excretion, which is indicative of excessive salt intake. This finding suggests that esaxerenone may be effective for patients with hypertension suspected to be caused by excessive salt intake; however, the *post hoc* analysis included a small number of patients from a larger clinical study. Therefore, evidence of the efficacy of esaxerenone in hypertensive patients with excessive salt intake remains insufficient, especially from a clinical setting.

The ENaK study set the urinary Na/K ratio and estimated 24-h urinary Na excretion as diagnostic markers to discriminate hypertensive patients with MR activation and hypertensive patients with excessive salt intake, respectively, and then exploratorily examined if baseline Na/K ratio and baseline estimated 24-h urinary Na excretion can predict the antihypertensive effect of esaxerenone.

Point of view

- Clinical relevance:
  The antihypertensive effect of esaxerenone was independent of the urinary sodium/potassium ratio and estimated 24-h urinary sodium excretion at baseline.
- Future direction:
  The results of this study were derived from a single spot urine collection and future studies using more accurate urine collection methods are needed to validate our findings.
- Consideration for the Asian population:
  Esaxerenone may contribute to BP control in Asian populations with diverse food cultures because it does not require consideration of individual sodium and potassium intake.

## Methods

### Study design

The ENaK study was an exploratory, open-label, interventional study conducted from October 2021 to June 2022 at 20 study sites in Japan. This study had a 4-week observation period and a 12-week treatment period (Figure S1). A list of participating institutions is provided in Table S1.

The present study was conducted in accordance with the principles of the Declaration of Helsinki and the Clinical Trials Act in Japan. The protocol was approved by the Certified Review Board of Hattori Clinic (CRB3180027). The study was prospectively registered at the Japan Registry of Clinical Trials (jRCTs031210273). All study participants provided written informed consent before enrollment.

### Treatment

Esaxerenone was orally administered once daily in accordance with the Japanese package insert, starting at a dose of 2.5 mg/day after a 4-week observation period. After the first 4 weeks of esaxerenone treatment, the dose could be gradually increased to 5 mg based on BP and serum K level monitoring, as shown in Figure S1. In patients with a creatinine-based estimated glomerular filtration rate (eGFR) 30 to <60 mL/min/1.73 m^2^ or with diabetes mellitus and albuminuria or proteinuria at baseline, the starting dose of esaxerenone was 1.25 mg/day. Patients who were undergoing treatment with antihypertensive agents other than esaxerenone continued their treatment without any changes in the dose or type of agent from the observation period to end of treatment (EOT).

Concomitant drugs that were prohibited from 4 weeks before starting treatment to the end of treatment were the following: antihypertensive drugs (e.g., angiotensin-converting enzyme inhibitors, α-blockers, β-blockers, αβ-blockers, vasodilators, or renin inhibitors), diuretics (e.g., thiazide, thiazide-like, loop, or K-sparing diuretics), MR blockers other than esaxerenone, angiotensin receptor neprilysin inhibitors, glycyrrhizin preparations, herbal medicines containing licorice, serum K inhibitors, and hyperkalemia-improving agents.

### Patients

Study participants who met all the following criteria were included in this study: age ≥20 years, prior use of antihypertensive agents (one ARB or one CCB) at the same dose for 4 weeks prior to study registration, and mean morning home systolic BP (SBP) ≥ 125 mmHg and/or diastolic BP (DBP) ≥ 75 mmHg measured with a brachial sphygmomanometer in the last 5 days prior to the registration date.

The main exclusion criteria were as follows: diagnosis of secondary hypertension (e.g., endocrine hypertension or hypertension due to a solitary kidney); hyperkalemia or serum K level >5.0 mEq/L; severe renal dysfunction (eGFR <30 mL/min/1.73 m^2^); severe hepatic dysfunction; history of myocardial infarction or heart failure requiring hospitalization, percutaneous coronary angioplasty, coronary artery bypass surgery, cerebral infarction, cerebral hemorrhage, subarachnoid hemorrhage, or transient ischemic attack within 12 weeks before obtaining consent; and those judged as inappropriate for this study by the study investigator.

### Outcome measurement

Home BP measurements were conducted daily by the patients themselves in the morning and at bedtime, and the average of the two measurements within the last 5 days before the patient’s visit was used. The same upper arm cuff sphygmomanometer (HCR-7501T, OMRON, Kyoto, Japan) was used throughout the study period. Home BP was automatically measured, and all data were collected electronically. Dietary salt intake was evaluated using a salt check sheet comprising 13 questions [34]. Spot urine samples were collected, and albumin and creatinine concentrations were measured in a central measurement laboratory (LSI Medience Corp., Tokyo, Japan) at baseline, Week 12, and discontinuation. Serum K and serum creatinine levels were measured at baseline; Weeks 2, 4, 6, 8, 10, and 12; and discontinuation at each study site. Plasma aldosterone concentration (PAC), plasma renin activity (PRA), serum N-terminal pro-brain natriuretic peptide (NT-proBNP), and urinary concentrations of Na, K, and creatinine were measured at baseline, Week 12, and discontinuation in the central measurement laboratory (LSI Medience Corp.). Other details on outcome measurement are included in the Supplementary methods.

### Endpoints

The primary efficacy endpoint was the change in morning home BP from baseline to EOT. The secondary efficacy endpoints were as follows: change in bedtime home and office BP from baseline to EOT; time course change in home and office BP during the study; achievement rate of target BP levels (home <125/75 mmHg and office <130/80 mmHg [2]) at Week 12; change and geometric percentage change in urinary albumin-creatinine ratio (UACR) and NT-proBNP from baseline; change from baseline in serum biomarkers (PAC and PRA) and urinary biomarkers (urinary Na, K, and Na/K ratio); and correlation between the change in BP and the urinary Na/K ratio, and the estimated 24-h urinary Na excretion. The safety endpoints were incidences of treatment-emergent adverse events (TEAEs), the incidence of serum K level ≥5.5 and ≥6.0 mEq/L, and time course changes and change from baseline in serum K and eGFR.

### Statistical analysis

For sample size determination, we assumed a change in sitting morning home BP ± SD of −10.0 ± 19.0 mmHg and −5.0 ± 11.0 mmHg for SBP and DBP, respectively, based on previous studies [26, 35]. Considering a statistical power of ≥90% for both SBP and DBP and 55 participants as the sample size, the significance level was set at *p* < 0.05 (two-sided). Thus, the target sample size was 60 participants each for the ARB and CCB subcohorts. Multiplicity adjustment was not performed for multiple evaluation groups and multiple time points.

Analyses were conducted for the total population and by type of basal antihypertensive agent (ARB and CCB subcohorts). No statistical comparisons were pre-specified between subcohorts per the study protocol.

For the change in BP from baseline to each measurement point, summary statistics and 95% confidence intervals (CIs) were calculated, and measurements were compared using the paired *t*-test. For BP, missing values at the EOT were imputed by the last observation carried forward method using data from Week 4 onward. The Clopper–Pearson method was used to calculate 95% CIs for the achievement rate of target BP levels. The paired *t*-test was used to calculate and compare 95% CIs for the change and geometric percentage change from baseline in UACR and NT-proBNP. Because UACR and NT-proBNP do not generally follow a normal distribution, logarithmic transformation was conducted before the statistical tests. For serum and urinary biomarkers, 95% CIs for the change from baseline were calculated. For the correlation between the change from baseline in BP at Week 12 and the urinary Na/K ratio at baseline, Pearson’s correlation coefficient was calculated using a single regression analysis with the change from baseline in BP at Week 12 as the explanatory variable and urinary Na/K ratio at baseline as the objective variable. The correlation between BP at Week 12 and estimated 24-h urinary Na excretion was evaluated in a similar manner, using the estimated 24-h urinary Na excretion as the objective variable (*post hoc* analysis). BP according to the Na/K ratio (by tertile) was evaluated in the full analysis set (FAS) as a pre-specified analysis and in the per protocol set (PPS) as a *post hoc* analysis. BP according to the 24-h Na excretion (by tertile) was evaluated in both the FAS and PPS as a *post hoc* analysis.

TEAEs were coded by System Organ Class and Preferred Term according to the Medical Dictionary for Regulatory Activities, version J.24.0. Statistical significance was set at 5% (two-sided). The statistical analyses were performed using Statistical Analysis System software version 9.4 (SAS Institute Inc., Cary, NC, USA).

Other details of the statistical analysis are included in the Supplementary methods.

## Results

### Patients

In total, 139 patients provided informed consent, among whom 126 met the eligibility criteria and were enrolled (ARB subcohort, *n* = 67; CCB subcohort, *n* = 59). All 126 patients were included in the FAS and safety analysis. The PPS included 121 patients (ARB subcohort, *n* = 62; CCB subcohort, *n* = 59). In total, 116 patients completed the study (ARB subcohort, *n* = 62; CCB subcohort, *n* = 54).

Table 1 summarizes the patient baseline demographic and clinical characteristics. In the total population, the mean age was 61.2 years and 52.4% of patients were male. Mean morning home, bedtime home, and office SBP/DBP was 136.7/88.0, 130.5/82.0, and 141.4/86.5 mmHg, respectively. Mean NT-proBNP level was 59.9 pg/mL; UACR level, 34.6 mg/gCr; serum K level, 4.3 mEq/L; eGFR, 74.8 mL/min/1.73 m^2^; and urinary Na/K ratio, 3.1. The estimated 24-h urinary Na excretion was 169.2 mEq/L; estimated daily salt intake, 10.0 g/day; and mean salt check sheet score, 13.8 points. Among the total population, 26.2% of patients had type 2 diabetes mellitus, and no patients had heart failure. Compared with the ARB subcohort, the CCB subcohort tended to be younger; have a higher PAC; lower PRA, NT-proBNP, and UACR; and have a longer history of hypertension at baseline. However, no statistical significance test was performed. The last dose of esaxerenone was 1.25, 2.5, and 5 mg/day in 7.9%, 65.1%, and 27.0% of patients in the total population, respectively. In the ARB and CCB subcohorts, 13.4% and 1.7% of patients, respectively, remained at a dose of 1.25 mg/day.

**Table 1:** Baseline patient characteristics (full analysis set)

Characteristics	Total *N* = 126	ARB subcohort *n* = 67	CCB subcohort *n* = 59
Sex, male	66 (52.4)	37 (55.2)	29 (49.2)
Age, years	61.2 ± 11.6	62.5 ± 12.3	59.8 ± 10.8
Weight, kg	68.0 ± 13.2	68.1 ± 13.6	67.8 ± 12.8
Body mass index, kg/m^2^	25.9 ± 4.2	26.0 ± 4.4	25.8 ± 4.0
Morning home SBP, mmHg	136.7 ± 12.1	137.3 ± 12.5	136.1 ± 11.7
Morning home DBP, mmHg	88.0 ± 9.0	87.7 ± 9.2	88.3 ± 8.8
Bedtime home SBP, mmHg	130.5 ± 13.6	130.7 ± 15.3	130.3 ± 11.7
Bedtime home DBP, mmHg	82.0 ± 9.1	81.4 ± 9.6	82.7 ± 8.6
Office SBP, mmHg	141.4 ± 15.2	141.1 ± 16.9	141.8 ± 13.1
Office DBP, mmHg	86.5 ± 10.8	84.4 ± 11.7	88.9 ± 9.1
NT-proBNP^a^, pg/mL	59.9 ± 61.5	72.6 ± 79.6	46.5 ± 28.3
<55	74 (63.2)	37 (61.7)	37 (64.9)
55 to <125	34 (29.1)	15 (25.0)	19 (33.3)
125 to <400	8 (6.8)	7 (11.7)	1 (1.8)
≥400	1 (0.9)	1 (1.7)	0 (0.0)
UACR^b^, mg/gCr	34.6 ± 130.8	44.1 ± 170.8	23.9 ± 58.5
<30	101 (81.5)	52 (78.8)	49 (84.5)
30 to <300	20 (16.1)	12 (18.2)	8 (13.8)
≥300	3 (2.4)	2 (3.0)	1 (1.7)
Serum K, mEq/L	4.3 ± 0.4	4.3 ± 0.3	4.2 ± 0.4
eGFR, mL/min/1.73 m^2^	74.8 ± 14.9	75.3 ± 16.8	74.2 ± 12.5
PAC, pg/mL	39.4 ± 26.8	36.2 ± 21.3	42.1 ± 30.7
PRA, ng/mL/h	4.2 ± 12.8	6.7 ± 17.0	1.3 ± 1.3
Duration of hypertension, years	6.0 ± 6.3	4.8 ± 5.4	7.7 ± 7.3
Complication			
T2DM	33 (26.2)	19 (28.4)	14 (23.7)
Dyslipidemia	74 (58.7)	40 (59.7)	34 (57.6)
Hyperuricemia	18 (14.3)	12 (17.9)	6 (10.2)
Heart failure	0 (0.0)	0 (0.0)	0 (0.0)
Urinary Na, mEq/L	108.0 ± 52.7	102.6 ± 50.1	114.2 ± 55.2
Urinary K, mEq/L	42.5 ± 23.5	41.3 ± 23.9	43.8 ± 23.0
Urinary Na/K ratio	3.1 ± 1.9	3.1 ± 2.0	3.1 ± 1.7
Estimated 24-h urinary Na excretion, mEq/day	169.2 ± 44.2	167.0 ± 41.7	171.6 ± 47.1
Estimated daily salt intake, g/day	10.0 ± 2.6	9.8 ± 2.5	10.1 ± 2.8
<6	4 (3.2)	1 (1.5)	3 (5.1)
6 to <10	63 (50.0)	36 (53.7)	27 (45.8)
≥10	59 (46.8)	30 (44.8)	29 (49.2)
Salt check sheet, points	13.8 ± 4.8	13.6 ± 4.7	14.1 ± 4.9
<9	19 (15.1)	11 (16.4)	8 (13.6)
9 to <14	43 (34.1)	25 (37.3)	18 (30.5)
≥14	64 (50.8)	31 (46.3)	33 (55.9)
Dose of esaxerenone at the end of treatment (last dose)			
1.25 mg	10 (7.9)	9 (13.4)	1 (1.7)
2.5 mg	82 (65.1)	41 (61.2)	41 (69.5)
5 mg	34 (27.0)	17 (25.4)	17 (28.8)

Data are *n* (%) or mean ± SD

^a^Population evaluated: Total, *N* = 117; ARB subcohort, *n* = 60; CCB subcohort, *n* = 57

^b^Population evaluated: Total, *N* = 124; ARB subcohort, *n* = 66; CCB subcohort, *n* = 58

*ARB* angiotensin receptor blocker, *CCB* calcium channel blocker, *DBP* diastolic blood pressure, *eGFR* creatinine-based estimated glomerular filtration rate, *K* potassium, *Na* sodium, *NT-proBNP* N-terminal pro-brain natriuretic peptide, *PAC* plasma aldosterone concentration, *PRA* plasma renin activity, *SBP* systolic blood pressure, *T2DM* type 2 diabetes mellitus, *UACR* urinary albumin-to-creatinine ratio

### BP-lowering effects of esaxerenone

**Fig. 1:**
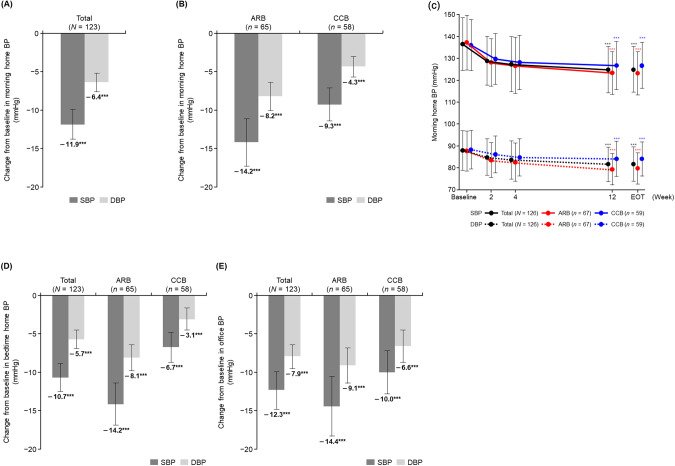
Change in morning home BP levels from baseline to EOT in the total population (**A**) and in the ARB and CCB subcohorts (**B**), time course changes from baseline in morning home BP (**C**), and change in bedtime home BP level (**D**) and office BP level (**E**) from baseline to EOT in the total population and in the ARB and CCB subcohorts (full analysis set). Data are mean (95% confidence interval) for panels A, B, D, and E. Data are mean ± SD for panel C. ***p < 0.001 vs baseline, paired *t*-test. *ARB* angiotensin receptor blocker, *BP* blood pressure, *CCB* calcium channel blocker, *DBP* diastolic blood pressure, *EOT* end of treatment, *SBP* systolic blood pressure

In the FAS, morning home SBP/DBP significantly decreased from baseline to EOT (−11.9 ± 10.9/ − 6.4 ± 6.8 mmHg, both *p* < 0.001) (Fig. 1A, Table S2). A significant reduction in SBP/DBP was also observed in both subcohorts from baseline to EOT (ARB subcohort, −14.2 ± 12.5/ − 8.2 ± 7.6 mmHg; CCB subcohort, −9.3 ± 8.2/ − 4.3 ± 5.0 mmHg; all *p* < 0.001) (Fig. 1B, Table S2). Morning home SBP/DBP decreased until Week 4 after starting esaxerenone treatment, and this BP reduction was maintained until Week 12 and EOT in the total population and both ARB and CCB subcohorts (Fig. 1C, Fig. S2A, Table S2).

Similar to the morning home BP, significant reductions were shown in bedtime home and office SBP/DBP (all *p* < 0.001) (Fig. 1D, E, Table S2). The trends of time course changes in bedtime home and office BP were also similar to that of morning home BP (Fig. S2B–E, and Table S2). Similar results were obtained in the PPS (Table S3).

The achievement rate of target morning home BP level at Week 12 was 12.1% in the total population, 16.1% in the ARB subcohort, and 7.4% in the CCB subcohort (Table S4). Similar trends were observed in the PPS (Table S5).

### Correlation of salt-related markers with BP

The correlation between each BP change from baseline to Week 12 and the baseline urinary Na/K ratio or baseline estimated 24-h urinary Na excretion are summarized in Fig. S3 and Table S6. No significant correlations were identified between each BP change and the urinary Na/K ratio or estimated 24-h urinary Na excretion at baseline. No significant correlations were also found in the PPS (Table S7).

**Fig. 2:**
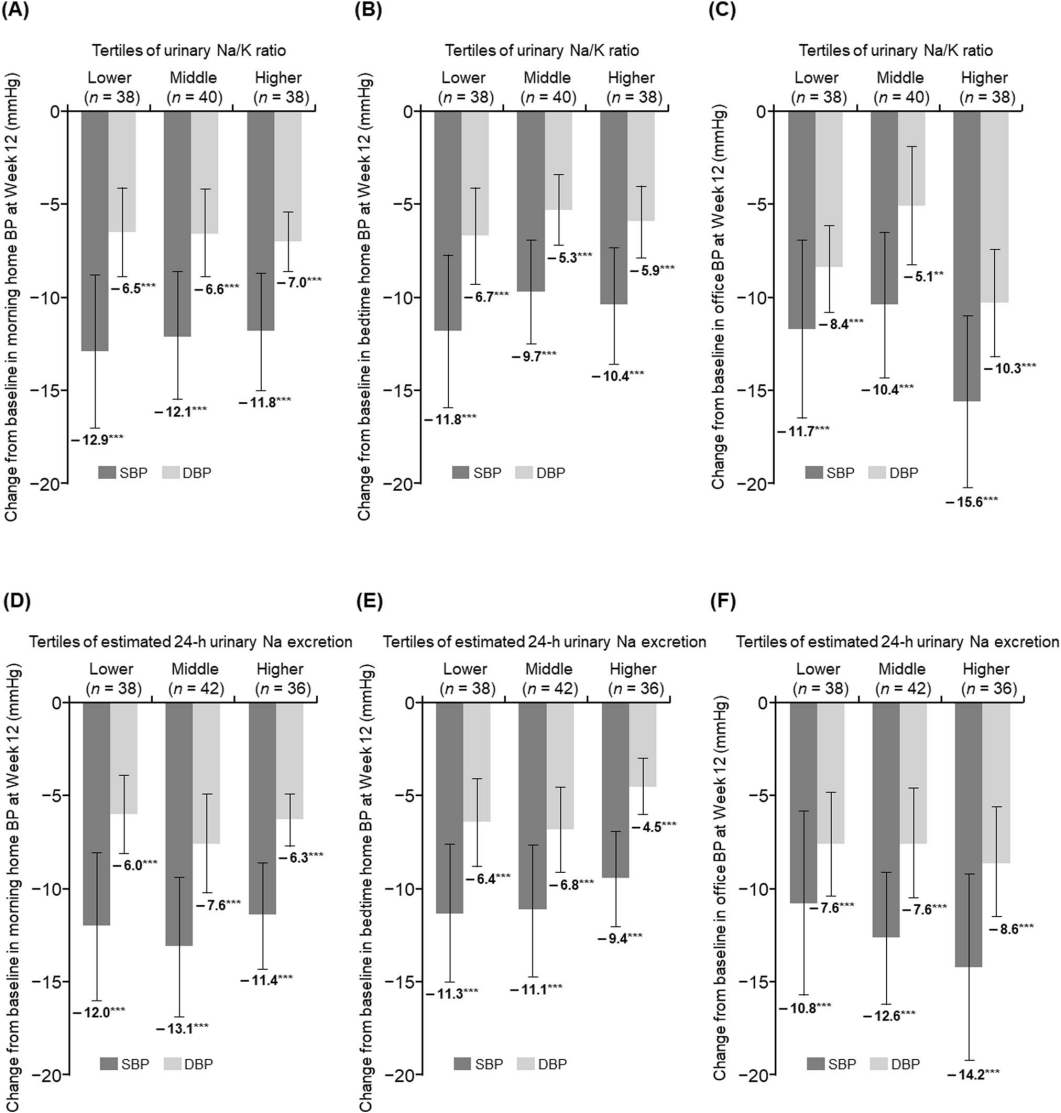
Change in morning home BP levels from baseline to Week 12 according to baseline urinary Na/K ratio (**A**, **B**, **C**: pre-specified analysis) and baseline 24-h urinary Na excretion (**D**, **E**, **F**: *post hoc* analysis) in the total population (**A**, **D**) and ARB (**B**, **E**) and CCB (**C**, **F**) subcohorts (full analysis set). Data are mean (95% confidence interval). **p < 0.01, ***p < 0.001 vs baseline, paired *t*-test. Tertiles of estimated 24-h urinary Na excretion (lower tertile, <142.5 mEq/day; middle tertile, 142.5 to 189.9 mEq/day; higher tertile, ≥189.9 mEq/day). *ARB* angiotensin receptor blocker, *BP* blood pressure, *CCB* calcium channel blocker, *DBP* diastolic blood pressure, *K* potassium, *Na* sodium, *SBP* systolic blood pressure

The change in each BP from baseline to Week 12 according to baseline urinary Na/K ratio (by tertiles) or baseline estimated 24-h urinary Na excretion (by tertiles) in the total population and ARB and CCB subcohorts are shown in Fig. 2, and Tables S8 and S9. In the total population, morning home BP significantly decreased from baseline to Week 12 irrespective of the baseline urinary Na/K ratio, and ranged from −11.8 to −12.9 mmHg in SBP and from −6.5 to −7.0 mmHg in DBP (all *p* < 0.001) (Fig. 2A and Table S8); similar decreases were shown in bedtime home and office BP (Fig. 2B, C, and Table S8). In addition, similar reductions were found in the ARB and CCB subcohorts as in the total population (Table S8).

A significant reduction in morning home BP was also observed regardless of baseline estimated 24-h urinary Na excretion (all *p* < 0.001) (Fig. 2D and Table S9). Similar trends were obtained in bedtime home and office BP in the total population and in each BP measurement in the ARB and CCB subcohorts (Fig. 2E, F, and Table S9). Similar trends to those in the FAS were obtained in the PPS (Tables S10 and S11).

### Effects of esaxerenone on the UACR and NT-proBNP

**Fig. 3:**
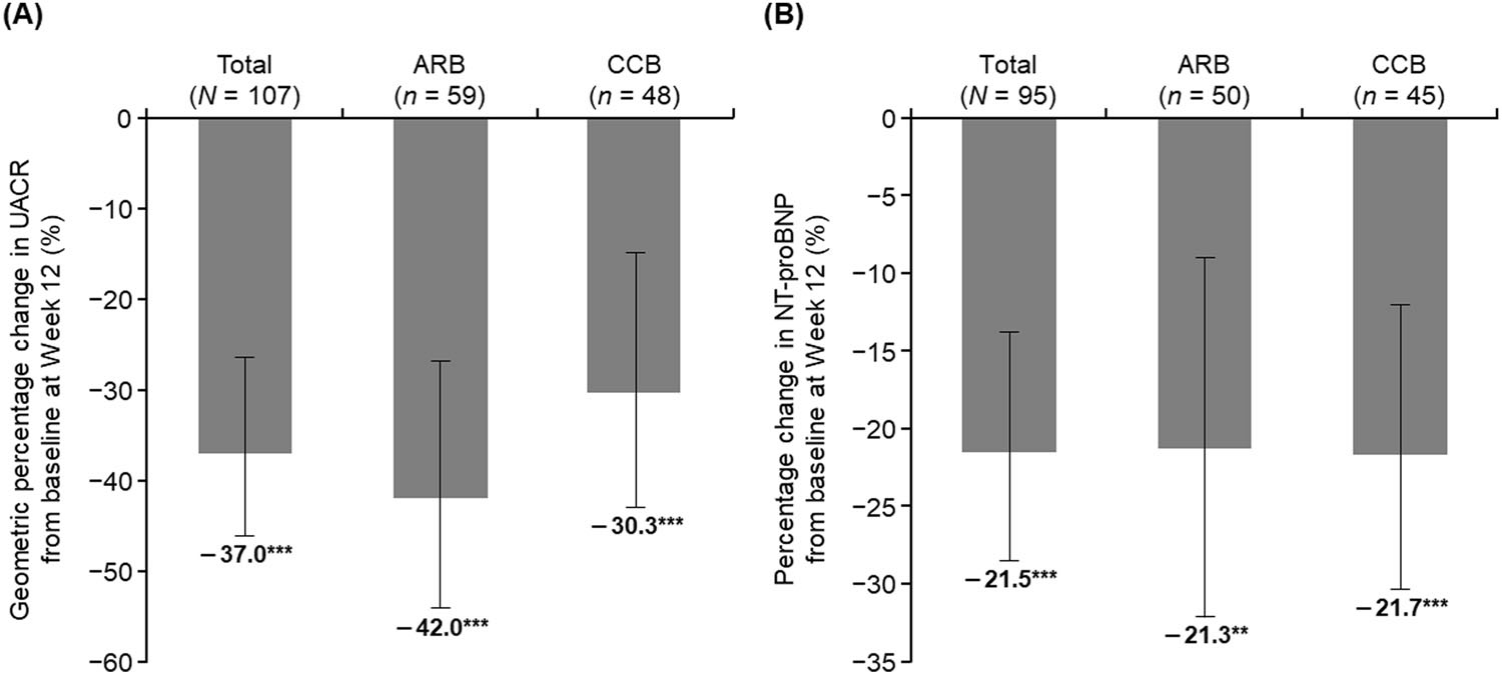
Geometric percentage change in UACR (**A**) and NT-proBNP (**B**) from baseline to Week 12 in the total population and ARB and CCB subcohorts (full analysis set). Data are mean (95% confidence interval). **p < 0.01, ***p < 0.001 vs baseline, paired *t*-test. *ARB* angiotensin receptor blocker, *CCB* calcium channel blocker, *NT-proBNP* N-terminal pro-brain natriuretic peptide, *UACR* urinary albumin-to-creatinine ratio

The UACR results are shown in Fig. 3A and Table S12. The UACR significantly decreased from baseline to Week 12 in the total population and ARB and CCB subcohorts (geometric percentage change, −37.0%, −42.0%, and −30.3% respectively; all *p* < 0.001) (Fig. 3A). The NT-proBNP significantly decreased from baseline to Week 12 in the total population and ARB and CCB subcohorts, and these reductions ranged from −21.3% to −21.7% (all *p* < 0.01) (Fig. 3B and Table S12). Similar reductions in UACR and NT-proBNP were also observed in the PPS (Table S13).

### Effects of esaxerenone on biomarkers

Serum and urinary biomarker data are shown in Table S14. In the total population, PAC and PRA increased with esaxerenone administration from baseline to Week 12 (mean ± SD change: PAC, 41.6 ± 44.0 pg/mL; PRA, 3.1 ± 14.5 ng/mL/h). Trends of these biomarkers were similar between the ARB and CCB subcohorts (mean ± SD change from baseline to Week 12: PAC, 30.3 ± 39.8 and 51.0 ± 45.4 pg/mL; PRA, 4.8 ± 19.7 and 1.2 ± 1.4 ng/mL/h, respectively) (Table S14). Regarding urinary biomarkers, urinary Na increased, but urinary K remained almost unchanged from baseline to Week 12 in the total population (mean ± SD change: Na, 14.6 ± 62.0 mEq/L; K, 0.9 ± 24.2 mEq/L), and the Na/K ratio slightly increased (0.3 ± 2.5) (Table S14). Similar trends were observed in the ARB and CCB subcohorts (Table S14). Similar results for serum and urinary biomarkers were also obtained in the PPS (Table S15).

### Safety

In the total population, 31.7% of patients had at least one TEAE (Table 2). Drug-related TEAEs were reported in 11 (8.7%) patients, of whom four (3.2%) discontinued the study treatment. Most TEAEs were mild or moderate. Serious TEAEs were reported in two (1.6%) patients, and these events (one ankle fracture and one glaucoma surgery and cataract operation) were not related to esaxerenone treatment. TEAEs related to serum K elevation were hyperkalemia in seven (5.6%) patients and blood potassium increased in one (0.8%) patient. Hyperkalemia was the most frequent TEAE, and three patients with hyperkalemia and one patient with blood potassium increased discontinued the study.

**Table 2:** Summary of TEAEs (safety analysis set)

Type of TEAE	Total *N* = 126	ARB subcohort *n* = 67	CCB subcohort *n* = 59
Any TEAEs	40 (31.7)	22 (32.8)	18 (30.5)
Serious TEAEs	2 (1.6)	2 (3.0)	0 (0.0)
Discontinued study treatment due to TEAEs	5 (4.0)	3 (4.5)	2 (3.4)
Drug-related TEAEs	11 (8.7)	6 (9.0)	5 (8.5)
Drug-related serious TEAEs	0 (0.0)	0 (0.0)	0 (0.0)
Discontinued study treatment due to drug-related TEAEs	4 (3.2)	2 (3.0)	2 (3.4)
Frequent TEAEs occurring in ≥2 patients			
Hyperkalemia	7 (5.6)	4 (6.0)	3 (5.1)
Diarrhea	3 (2.4)	3 (4.5)	0 (0.0)
Cystitis	2 (1.6)	0 (0.0)	2 (3.4)
Gastroenteritis	2 (1.6)	1 (1.5)	1 (1.7)
Nasopharyngitis	2 (1.6)	2 (3.0)	0 (0.0)
Gastritis	2 (1.6)	1 (1.5)	1 (1.7)
Pyrexia	2 (1.6)	2 (3.0)	0 (0.0)
Blood potassium increased^a^	1 (0.8)	1 (1.5)	0 (0.0)
Drug-related TEAEs			
Hyperkalemia	6 (4.8)	3 (4.5)	3 (5.1)
Dizziness postural	1 (0.8)	1 (1.5)	0 (0.0)
Constipation	1 (0.8)	0 (0.0)	1 (1.7)
Rash	1 (0.8)	0 (0.0)	1 (1.7)
Blood potassium increased	1 (0.8)	1 (1.5)	0 (0.0)
Blood pressure decreased	1 (0.8)	1 (1.5)	0 (0.0)

Data are *n* (%). MedDRA/J version 24.0

^a^Blood potassium increased is included in this table, although only reported in one patient, as it is a known adverse event of mineralocorticoid receptor blockers

*ARB* angiotensin receptor blocker, *CCB* calcium channel blocker, *MedDRA* Medical Dictionary for Regulatory Activities, *TEAE* treatment-emergent adverse event

**Fig. 4:**
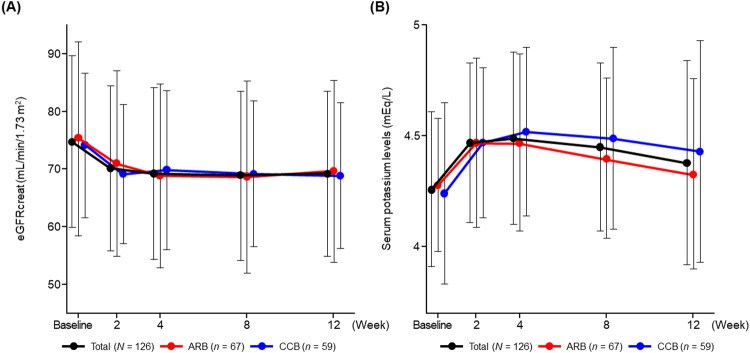
Time course changes in eGFR (**A**) and serum K levels (**B**) during the study period in the total population and ARB and CCB subcohorts (safety analysis set). Data are mean ± SD. *ARB* angiotensin receptor blocker, *CCB* calcium channel blocker, *eGFR* creatinine-based estimated glomerular filtration rate, *K* potassium

The eGFR decreased over first 4 weeks (mean ± SD change from baseline, −5.3 ± 8.2 mL/min/1.73 m^2^) and remained constant until Week 12 (mean ± SD change from baseline, −5.8 ± 8.0 mL/min/1.73 m^2^) (Figs. 4A, S4A). A similar trend of reduction in the eGFR was observed in the ARB and CCB subcohorts (mean change from baseline to Week 12: −6.8 ± 8.5 and −4.7 ± 7.4 mL/min/1.73 m^2^, respectively).

Serum K levels increased over the first 4 weeks after starting esaxerenone treatment, and then gradually decreased up to Week 12 in the total population and both subcohorts (Fig. 4B). The change in serum K was 0.2 ± 0.4 mEq/L at Week 4 and 0.1 ± 0.5 mEq/L at Week 12 (Fig. S4B). Similar trends in serum K were observed in the ARB and CCB subcohorts (mean ± SD change from baseline to Week 12: 0.1 ± 0.4 and 0.2 ± 0.6 mEq/L, respectively). In the total population, nine (7.1%) and two (1.6%) patients had serum K levels ≥5.5 and ≥6.0 mEq/L, respectively (Table S16).

## Discussion

In the ENaK study, we examined the correlation between the antihypertensive effect of esaxerenone and the baseline urinary Na/K ratio and estimated 24-h urinary Na excretion in hypertensive patients. The administration of esaxerenone significantly lowered morning home BP, as well as bedtime home and office BP, in patients whose hypertension was inadequately controlled with an ARB or a CCB. The antihypertensive effect of esaxerenone was not affected by the urinary Na/K ratio or estimated 24-h urinary Na excretion at baseline. The organ-protective markers, UACR and NT-proBNP levels, were also significantly reduced with esaxerenone. Decreases in the eGFR and elevations in serum K levels were observed, but the change over time and the amount of change were similar to those seen in previous clinical studies [25, 26, 36]. No new TEAEs or drug-related TEAEs requiring caution were observed.

The aim of this study was to investigate the correlation between the antihypertensive effect of esaxerenone and the baseline urinary Na/K ratio and estimated 24-h urinary Na excretion in hypertensive patients. Although the antihypertensive effect of esaxerenone could be confirmed in this study, morning home, bedtime home and office BPs were significantly reduced from baseline values, regardless of each tertile of baseline urinary Na/K ratio and estimated 24-h urinary Na excretion, and no correlation was confirmed. The results of this study, in which the BP-lowering effects of esaxerenone were consistently significant regardless of the estimated 24-h urinary Na excretion at baseline, differ from the results of previous studies of esaxerenone and eplerenone. A previous substudy of a long-term phase 3 study suggested that the antihypertensive effect of esaxerenone monotherapy was stronger in patients with higher vs lower Na excretion at baseline [33]. In the EVALUATE study, eplerenone also exhibited strong antihypertensive and UACR-reducing activities in the highest tertile of the baseline estimated 24-h urinary Na excretion [20]. The reason for this discrepancy may be differences in patient background factors (e.g., distribution range of the baseline estimated 24-h urinary Na excretion) between the studies. The mean estimated 24-h urinary Na excretion at baseline was 169.2 ± 44.2 mEq/day in the present study, 212.8 ± 97.4 mEq/day in the phase 3 substudy [33], and 219.5 ± 66.1 mEq/day in the eplerenone group of the EVALUATE study [20, 37]. In addition, the median urinary Na excretion in the phase 3 substudy was 193.8 mEq/day, but only 37 of 126 patients in the ENaK study exceeded this median. Only eight of the 126 patients in the ENaK study were classified into the highest tertile of the EVALUATE study ( > 235 mEq/day), whereas 83 of 126 patients in the ENaK study were classified in the lowest tertile of the EVALUATE study (<189 mEq/day). Thus, the distribution of the estimated 24-h Na excretion in the ENaK study was biased toward lower values than in previous studies and should be examined in a study incorporating patients with higher Na excretion to verify the association between Na excretion and the antihypertensive effect of esaxerenone.

The urinary Na/K ratio is not only one of the main indicators of salt loading [38, 39], but it can also be an indirect indicator of MR activation, since MRs promote Na reabsorption and K excretion in the kidneys [23, 24]. In other words, the Na/K ratio could be a good indicator of efficacy for patients best suited for MR blocker administration. However, the antihypertensive effect of esaxerenone was not affected by the baseline Na/K ratio in the present study. One possible reason for this is the conditions under which the spot urine was collected. The previous reports have shown that fasting time before the measurement, eGFR, and seasonal variations affect the Na/K ratio [38]; thus, the collection of six random daytime casual urine samples on separate days is necessary to accurately determine an individual’s Na/K level [40]. In the ENaK study, the Na/K ratio was calculated from a single spot urine collected just before the start of esaxerenone administration. In the future, the relevance of the antihypertensive effect of esaxerenone under conditions that would allow measuring the accurate Na/K ratio of each patient should be investigated.

Compared with office BP, home BP is a better predictor of cerebrovascular risk, and morning home BP is particularly important, as mentioned in the 2019 Japanese Society of Hypertension guidelines [2, 41, 42]. In the ENaK study, esaxerenone significantly reduced morning home BP in hypertensive patients inadequately controlled with an ARB or a CCB. This result was consistent with that of the EX-DKD study, the only published study examining home BP with the 12-week administration of esaxerenone in hypertensive patients with diabetes and eGFR 30–60 mL/min/1.73 m^2^, inadequately controlled with a renin–angiotensin system (RAS) inhibitor or RAS inhibitor plus CCB (ENaK study at EOT, −11.9/ − 6.4 mmHg; EX-DKD study at EOT, −11.6/ − 5.2 mmHg) [36]. The antihypertensive effects of esaxerenone on bedtime home BP and office BP were also similar between the ENaK study and the EX-DKD study (bedtime home SBP/DBP change at EOT: ENaK study, −10.7/ − 5.7 mmHg; EX-DKD study, −10.0/ − 4.4 mmHg; office SBP/DBP change at EOT: ENaK study, −12.3/ − 7.9 mmHg; EX-DKD study, −11.5/ − 5.2 mmHg) [36]. The antihypertensive effect on office BP in previous phase 3 clinical studies in patients with essential hypertension was also comparable to the results obtained in this study [25, 26]. These results suggest that esaxerenone has a consistent and favorable home BP and office BP-lowering effect, independent of patient background.

Although no significant difference tests were performed, the antihypertensive effect of esaxerenone both in home and office BP appeared to be stronger in the ARB vs CCB subcohort. Compared with the CCB subcohort, patients in the ARB subcohort tended to have lower PAC, higher PRA, and a shorter history of hypertension at baseline. However, this patient background information is insufficient for our estimates of differences in the antihypertensive effect of esaxerenone between subcohorts. A pooled analysis of seven phase 3 studies of esaxerenone suggested that factors such as female sex, lower body weight, lower PAC, and lower UACR potentiate the office BP-lowering effects of esaxerenone [43], but in the ENaK study, the differences in these factors between the subcohorts appeared to be unremarkable. It remains to be clarified whether the antihypertensive effect of esaxerenone is stronger when combined with an ARB than with a CCB, and if this is true, what background factors are responsible for this difference.

Both UACR and NT-proBNP levels decreased significantly with esaxerenone treatment in the present study. This organ-protective effect of esaxerenone in lowering UACR and NT-proBNP has been confirmed in phase 3 studies [26, 29, 31, 32] and a post-marketing clinical study, EX-DKD [36]. In this study, most patients (101 patients, 81.5%) were classified as having normal albuminuria (<30 mg/gCr), and only 23 patients (18.5%) had micro- (30–300 mg/gCr) and macroalbuminuria (≥300 mg/gCr). Despite this uneven patient distribution, the 37.0% reduction in UACR (pre-treatment: 34.6 mg/gCr; Week 12 post-treatment: 17.0 mg/gCr) suggests that esaxerenone may prevent renal function deterioration associated with increased albuminuria. NT-proBNP, which showed a distribution with most normal ranges similar to UACR (<55 pg/mL: 74 patients, 63.2%; ≥55 pg/mL: 43 patients, 36.8%), was also significantly suppressed by esaxerenone. Esaxerenone may be a treatment option that reduces the risk of both cardiovascular and renal events, considering the role of UACR as a risk factor of cardiac and renal events [44–46] and NT-proBNP as a predictor of cardiovascular risk and kidney damage [47–49].

The dual nature of esaxerenone as an antihypertensive and organ-protective agent has been demonstrated by a subanalysis of a phase 3 study, the ESAX-DN study. This analysis reported that only <30% of the UACR-lowering effect of esaxerenone was attributed to its antihypertensive effect, i.e., the reduction in circulating and renal blood flow associated with a decrease in SBP and eGFR, and the remaining 70% was attributed to direct MR inhibition [50].

In the present study, the incidences of TEAEs and drug-related TEAEs were 31.7% and 8.7%, respectively, and were similar to those reported in a series of phase 3 studies and a post-marketing clinical study of esaxerenone with hypertensive patients with various patient background characteristics [25–32, 36]. In addition, no new safety concerns were identified, although some caution is warranted regarding serum K elevation. Serum K elevation is a well-known adverse effect of MR blockers [51], and hyperkalemia was the most frequent TEAE in this study. However, the incidences of TEAEs related to serum potassium elevation (6.4%) and that of serum potassium level ≥5.5 mEq/L and ≥6.0 mEq/L (7.1% and 1.6%, respectively) were similar to those reported in phase 3 studies in patients with essential hypertension [25, 26]. Concomitant use of RAS inhibitors and MR blocker has been reported to be one of the risk factors for serum K elevation [51]. But in this study, the incidence of TEAEs and drug-related TEAEs associated with serum K elevation, and the time course change in serum K levels did not differ between the ARB and CCB subcohorts, and concomitant use of ARB and esaxerenone did not cause serum K elevation.

In the present study, the eGFR decreased during the first 4 weeks of treatment and then remained constant until Week 12 in the total population and the ARB and CCB subcohorts. The mean eGFR change from baseline to Week 12 was −5.8 mL/min/1.73 m^2^ in the total population. These trends in changes over time and mean change in eGFR were consistent with those reported previously in hypertensive patients with various complications who were treated with esaxerenone [25–32, 36].

### Study limitations

In the present study, urinary biomarkers were calculated using a single spot urine collection, but not using multiple random urine collections on separate days or 24-h urine collections. This may have affected the analysis of correlations between urinary biomarkers and antihypertensive effect. Recently, the World Hypertension League, the International Society of Hypertension, and other organizations published a consensus document stating that studies using spot urine samples should not be conducted [52]. Future studies using more accurate urine collection methods are needed to validate the results of this study. In addition, the estimated daily salt intake was calculated on the basis of urinary Na excretion in this study. To enable more accurate interpretation of the results, another salt intake measurement index independent of Na excretion might be needed. There were other limitations related to the exploratory nature of the study (no adjustments for multiplicity) and the open-label, single-arm study design (no active comparator). Differences between subcohorts were not statistically compared. In this study, PAC and PRA (indicators of MR activity inhibition [53]) increased with the decrease in BP, and a ‘placebo-induced’ antihypertensive effect was not deemed to be the primary contributor for BP reduction. In addition, the habit of regularly measuring BP at home during the 4-week observation period and the 3-month study period may have improved BP control in the patients. Furthermore, ARBs alter the slope of Guyton’s pressure-natriuretic curve [54] and may have modified the correlation between antihypertensive effect of esaxerenone and urinary Na excretion in this study; however, the effect on Na excretion of ARBs administered more than 4 weeks before the start of esaxerenone administration would have reached a steady state and might have had a limited impact on the study results. Finally, the generalizability of this study is limited to the Japanese population.

### Perspectives in Asia

There is increasing epidemiological evidence of an association between the urinary Na/K ratio and BP or cardiovascular events [55, 56]. However, due to differences in food culture, Asians have not only a higher salt intake than other regions, but also differences in their consumption of potassium-rich foods such as vegetables and fruits [57, 58]. Therefore, it is difficult to clearly define a cutoff value for the Na/K ratio, and no global consensus has been reached.

The antihypertensive effect of esaxerenone independent of urinary sodium/potassium ratio at baseline is expected to contribute to achieving the goal of “zero” cardiovascular events in Asia [59].

## Conclusions

Esaxerenone significantly reduced morning home BP as well as bedtime home and office BP, and also significantly decreased UACR and NT-proBNP in patients with essential hypertension inadequately controlled with an ARB or a CCB. These effects were consistent regardless of concomitant use of antihypertensive drugs (ARB and CCB) and were achieved without clinically relevant serum K elevation or any new safety concerns. This antihypertensive effect of esaxerenone was independent of the urinary Na/K ratio and estimated 24-h urinary Na excretion at baseline.

### Supplementary information

Supplementary Materials

Supplementary Tables

Supplementary Figures

## Data Availability

The anonymized data underlying the results presented in this manuscript may be made available to researchers upon submission of a reasonable request to the corresponding author. The decision to disclose the data will be made by the corresponding author and the funder, Daiichi Sankyo Co., Ltd. Data disclosure can be requested for 36 months from article publication.
