# Supplementary material for: Exploratory study on the relationship between urinary sodium/potassium ratio, salt intake, and the antihypertensive effect of esaxerenone: the ENaK Study
Source: Hypertens Res. 2024 Jan 11;47(4):835–48. doi: 10.1038/s41440-023-01519-0 (PMC10994843; doi:10.1038/s41440-023-01519-0)
Supplement: Supplementary file 1 — Supplementary Materials [file 41440_2023_1519_MOESM1_ESM.docx]

# Supplementary Materials

**Supplementary methods**. Additional details of the study methods

## BP measurements

In the morning, blood pressure (BP) measurements were conducted within 1 h after waking up, after urination, and before breakfast, medication, and caffeine intake. At bedtime, BP measurements were conducted more than 1 h after bathing, smoking, drinking, or caffeine intake. Office BP measurements were conducted two times at each visit (at baseline, Weeks 4 and 12, and discontinuation). The average of the two measurements was recorded. If the dose of esaxerenone was increased at Weeks 4 and 8, the home BP was also measured at Weeks 6 and 10, and in patients with a starting dose of esaxerenone of 1.25 mg/day, the home and office BP were measured at Week 8.

## Measurement of other outcomes

Each question on the salt check sheet was scored from 0 to 2 or 3 for a total of 35 points. Dietary salt intake was characterized as low (0–8 points), medium (9–13 points), high (14–19 points), or very high (≥20 points). Patients were asked to complete the salt check sheet at the start of the observation period.

The following formula was used to calculate the urinary albumin-creatinine ratio (UACR): UACR (mg/gCr) = urinary albumin (µg/mL)/urinary creatinine (mg/dL) × 100. The following formula was used to calculate the estimated glomerular filtration rate: 194 × serum creatinine^−1.094^ × age^−0.287^, multiplied by 0.739 for female patients. Serum potassium (K) measurements at Weeks 4 and 8 were used to determine if the esaxerenone dose should be increased. If the dose was escalated at Weeks 4 and 8, safety was confirmed by serum K measurements at Weeks 6 and 10, respectively. Plasma aldosterone concentration and plasma renin activity were measured with intervals of ≥2 h after meals and after resting in the supine position for at least 30 minutes. Urinary sodium (Na)/K ratios were calculated from the urinary concentrations of Na and K. Estimated 24-h urinary creatinine, estimated 24-h urinary Na excretion, and estimated daily salt intake were calculated using the following formula: Estimated 24-h urinary creatinine excretion (mg/day) = (body weight, kg) × 14.89 + (height, cm) × 16.14 − age × 2.043 − 2244.45. Estimated 24-h urinary Na excretion (mEq/day) = 21.98 × [(urinary Na, mEq/L) / (urinary creatinine, mg/dL) / 10 × (estimated 24-h urinary creatinine excretion, mg/day)] × 0.392. Estimated daily salt intake (g/day) = (estimated 24-h urinary Na excretion, mEq/day) / 17.

## Statistical analysis

The full analysis set (FAS) was used to evaluate efficacy endpoints and was defined as all patients who met the inclusion criteria, received at least one dose of esaxerenone, and had at least one efficacy measurement recorded. The safety analysis set was used to evaluate safety endpoints and was defined as all enrolled patients who took at least one dose of esaxerenone. The per protocol set was defined as FAS patients who adhered to the esaxerenone package insert.
