# Supplementary material for: Exploratory study on the relationship between urinary sodium/potassium ratio, salt intake, and the antihypertensive effect of esaxerenone: the ENaK Study
Source: Hypertens Res. 2024 Jan 11;47(4):835–48. doi: 10.1038/s41440-023-01519-0 (PMC10994843; doi:10.1038/s41440-023-01519-0)
Supplement: Supplementary file 2 — Supplementary Tables [file 41440_2023_1519_MOESM2_ESM.docx]

# Supplementary Materials

# Supplementary Tables

**Table S1.** List of participating institutions and representative physicians^a^

| **Institution** | | **Representative physicians** | |
| --- | --- | --- | --- |
| Katsuya Clinic | | Tomohiro Katsuya  (Principal investigator) | |
| Asamoto Internal Medicine Clinic | | Arata Iwasaki | |
| Hatta Medical Clinic | | Tsuguru Hatta | |
| Hirano Clinic^b^ | | Shigeru Hirano, Kunio Hirano | |
| Ikushima Clinic | | Masashi Ikushima | |
| Inobe Funai Clinic | | Yoshito Inobe | |
| Jomo Ohashi Clinic | | Tetsuya Shigehara | |
| Kashinoki Internal Medicine | | Hajime Ishii | |
| Kato Clinic of Internal Medicine | | Mitsutoshi Kato | |
| Koseikai Clinic | | Izuru Masuda | |
| Maebashi Hirosegawa Clinic | | Shintaro Yano | |
| Medical Corporation Association Koukeikai Sugiura Clinic | | Toshiyuki Sugiura | |
| Medical Corporation Shirayurikai Swing Nozaki Clinic | | Minoru Nozaki | |
| Nishikawa Clinic | | Tetsuo Nishikawa | |
| Noguchi Medical Clinic | | Yuichi Noguchi | |
| Olive Takamatsu Medical Clinic | | Toshiki Fukui | |
| Takeuchi Clinic | | Motoshi Takeuchi | |
| Uchiyama Clinic | | Kazuaki Uchiyama | |
| Usaka Clinic | | Satoshi Kagitani | |
| Yatagai Clinic | Shigeo Yatagai | |  |

^a^ Institutions are listed in alphabetical order except for the principal investigator.

^b^ In this institution, there was a change in the principal investigator during the course of the study.

**Table S2.** Change from baseline in BP (full analysis set)

| **BP** | **Total**  ***N* = 126** | | **ARB** **subcohort**  ***n* = 67** | | | **CCB subcohort**  ***n* = 59** | |
| --- | --- | --- | --- | --- | --- | --- | --- |
|  | **SBP, mmHg** | **DBP, mmHg** | **SBP, mmHg** | **DBP, mmHg** | **SBP, mmHg** | | **DBP, mmHg** |
| Morning home BP |  |  |  |  |  | |  |
| Baseline, *n* | 126 | 126 | 67 | 67 | 59 | | 59 |
| Mean ± SD | 136.7 ± 12.1 | 88.0 ± 9.0 | 137.3 ± 12.5 | 87.7 ± 9.2 | 136.1 ± 11.7 | | 88.3 ± 8.8 |
| Week 2, *n* | 123 | 123 | 65 | 65 | 58 | | 58 |
| Mean ± SD | 129.0 ± 11.2 | 84.9 ± 8.3 | 128.2 ± 10.8 | 83.6 ± 8.0 | 129.8 ± 11.6 | | 86.2 ± 8.4 |
| Change from baseline | −7.9 ± 7.8 | −3.3 ± 4.7 | −9.3 ± 8.2 | −4.3 ± 4.7 | −6.3 ± 7.1 | | −2.2 ± 4.5 |
| Week 4, *n* | 121 | 121 | 64 | 64 | 57 | | 57 |
| Mean ± SD | 127.4 ± 12.6 | 83.6 ± 8.7 | 126.8 ± 12.8 | 82.6 ± 8.8 | 128.2 ± 12.4 | | 84.8 ± 8.4 |
| Change from baseline | −9.4 ± 8.2 | −4.5 ± 4.9 | −10.8 ± 9.2 | −5.3 ± 5.2 | −7.9 ± 6.5 | | −3.7 ± 4.4 |
| Week 12, *n* | 116 | 116 | 62 | 62 | 54 | | 54 |
| Mean ± SD | 124.9 ± 10.5 | 81.7 ± 7.9 | 123.3 ± 9.8 | 79.5 ± 7.2 | 126.8 ± 11.0 | | 84.2 ± 8.1 |
| Change from baseline | −12.3 ± 10.9*** | −6.7 ± 6.7*** | −14.4 ± 12.6*** | −8.5 ± 7.4*** | −9.8 ± 8.2*** | | −4.6 ± 4.9*** |
| EOT, *n* | 123 | 123 | 65 | 65 | 58 | | 58 |
| Mean ± SD | 125.0 ± 10.4 | 81.8 ± 7.8 | 123.3 ± 9.9 | 79.7 ± 7.2 | 126.8 ± 10.7 | | 84.1 ± 7.9 |
| Change from baseline | −11.9 ± 10.9*** | −6.4 ± 6.8*** | −14.2 ± 12.5*** | −8.2 ± 7.6*** | −9.3 ± 8.2*** | | −4.3 ± 5.0*** |
| Bedtime home BP |  |  |  |  |  | |  |
| Baseline, *n* | 126 | 126 | 67 | 67 | 59 | | 59 |
| Mean ± SD | 130.5 ± 13.6 | 82.0 ± 9.1 | 130.7 ± 15.3 | 81.4 ± 9.6 | 130.3 ± 11.7 | | 82.7 ± 8.6 |
| Week 2, *n* | 123 | 123 | 65 | 65 | 58 | | 58 |
| Mean ± SD | 124.3 ± 12.4 | 79.5 ± 8.9 | 121.7 ± 12.7 | 76.8 ± 8.1 | 127.3 ± 11.4 | | 82.4 ± 8.8 |
| Change from baseline | −6.5 ± 9.0 | −2.8 ± 5.7 | −9.6 ± 9.5 | −5.0 ± 5.7 | −3.1 ± 6.9 | | −0.3 ± 4.6 |
| Week 4, *n* | 121 | 121 | 64 | 64 | 57 | | 57 |
| Mean ± SD | 122.4 ± 12.9 | 78.0 ± 8.9 | 120.4 ± 13.9 | 75.7 ± 8.8 | 124.7 ± 11.4 | | 80.6 ± 8.4 |
| Change from baseline | −8.5 ± 9.2 | −4.3 ± 5.6 | −11.0 ± 10.3 | −6.1 ± 6.0 | −5.8 ± 7.0 | | −2.4 ± 4.4 |
| Week 12, *n* | 116 | 116 | 62 | 62 | 54 | | 54 |
| Mean ± SD | 120.3 ± 11.4 | 76.4 ± 8.9 | 117.4 ± 11.4 | 73.5 ± 8.7 | 123.7 ± 10.4 | | 79.7 ± 8.0 |
| Change from baseline | −10.6 ± 10.3*** | −6.0 ± 6.6*** | −13.9 ± 11.2*** | −8.2 ± 6.8*** | −6.9 ± 7.6*** | | −3.4 ± 5.4*** |
| EOT, *n* | 123 | 123 | 65 | 65 | 58 | | 58 |
| Mean ± SD | 120.2 ± 11.3 | 76.6 ± 8.7 | 117.2 ± 11.5 | 73.8 ± 8.6 | 123.6 ± 10.1 | | 79.7 ± 7.7 |
| Change from baseline | −10.7 ± 10.3*** | −5.7 ± 6.7*** | −14.2 ± 11.2*** | −8.1 ± 6.8*** | −6.7 ± 7.5*** | | −3.1 ± 5.5*** |
| Office BP |  |  |  |  |  | |  |
| Baseline, *n* | 126 | 126 | 67 | 67 | 59 | | 59 |
| Mean ± SD | 141.4 ± 15.2 | 86.5 ± 10.8 | 141.1 ± 16.9 | 84.4 ± 11.7 | 141.8 ± 13.1 | | 88.9 ± 9.1 |
| Week 4, *n* | 121 | 121 | 64 | 64 | 57 | | 57 |
| Mean ± SD | 130.9 ± 13.8 | 81.2 ± 11.6 | 130.1 ± 14.7 | 79.2 ± 12.5 | 131.7 ± 12.8 | | 83.4 ± 10.1 |
| Change from baseline | −10.5 ± 13.8 | −5.4 ± 8.9 | −11.3 ± 15.9 | −5.4 ± 9.6 | −9.5 ± 11.0 | | −5.5 ± 8.2 |
| Week 12, *n* | 116 | 116 | 62 | 62 | 54 | | 54 |
| Mean ± SD | 128.8 ± 12.6 | 78.8 ± 11.3 | 127.2 ± 13.8 | 75.8 ± 10.8 | 130.7 ± 10.9 | | 82.3 ± 10.9 |
| Change from baseline | −12.5 ± 13.7*** | −7.9 ± 8.8*** | −14.5 ± 15.9*** | −8.8 ± 9.3*** | −10.3 ± 10.3*** | | −6.8 ± 8.2*** |
| EOT, *n* | 123 | 123 | 65 | 65 | 58 | | 58 |
| Mean ± SD | 128.9 ± 12.8 | 78.7 ± 11.3 | 126.8 ± 13.6 | 75.4 ± 10.9 | 131.2 ± 11.4 | | 82.3 ± 10.7 |
| Change from baseline | −12.3 ± 13.6*** | −7.9 ± 8.7*** | −14.4 ± 15.6*** | −9.1 ± 9.2*** | −10.0 ± 10.7*** | | −6.6 ± 8.0*** |

****p* <0.001 vs baseline, paired *t*-test.

*p*-values were not calculated for changes from baseline to Week 4.

*ARB* angiotensin receptor blocker, *BP* blood pressure, *CCB* calcium channel blocker, *DBP* diastolic blood pressure, *EOT* end of treatment, *SBP* systolic blood pressure.

**Table S3.** Change from baseline in BP (per protocol set)

| **BP** | **Total**  ***N* = 121** | | **ARB** **subcohort**  ***n* = 62** | | | **CCB** **subcohort**  ***n* = 59** | |
| --- | --- | --- | --- | --- | --- | --- | --- |
|  | **SBP, mmHg** | **DBP, mmHg** | **SBP, mmHg** | **DBP, mmHg** | **SBP, mmHg** | | **DBP, mmHg** |
| Morning home BP |  |  |  |  |  | |  |
| Baseline, *n* | 121 | 121 | 62 | 62 | 59 | | 59 |
| Mean ± SD | 136.7 ± 12.2 | 88.1 ± 8.9 | 137.3 ± 12.7 | 87.9 ± 9.0 | 136.1 ± 11.7 | | 88.3 ± 8.8 |
| Week 2, *n* | 119 | 119 | 61 | 61 | 58 | | 58 |
| Mean ± SD | 128.8 ± 11.3 | 84.8 ± 8.4 | 127.9 ± 11.1 | 83.4 ± 8.1 | 129.8 ± 11.6 | | 86.2 ± 8.4 |
| Change from baseline | −7.9 ± 7.8 | −3.4 ± 4.7 | −9.5 ± 8.1 | −4.5 ± 4.5 | −6.3 ± 7.1 | | −2.2 ± 4.5 |
| Week 4, *n* | 117 | 117 | 60 | 60 | 57 | | 57 |
| Mean ± SD | 127.3 ± 12.7 | 83.6 ± 8.8 | 126.5 ± 12.9 | 82.4 ± 9.1 | 128.2 ± 12.4 | | 84.8 ± 8.4 |
| Change from baseline | −9.4 ± 8.2 | −4.6 ± 4.9 | −10.9 ± 9.4 | −5.4 ± 5.2 | −7.9 ± 6.5 | | −3.7 ± 4.4 |
| Week 12, *n* | 112 | 112 | 58 | 58 | 54 | | 54 |
| Mean ± SD | 125.0 ± 10.5 | 81.7 ± 8.1 | 123.3 ± 9.8 | 79.4 ± 7.4 | 126.8 ± 11.0 | | 84.2 ± 8.1 |
| Change from baseline | −12.1 ± 10.8*** | −6.6 ± 6.6*** | −14.2 ± 12.5*** | −8.6 ± 7.3*** | −9.8 ± 8.2*** | | −4.6 ± 4.9*** |
| EOT, *n* | 119 | 119 | 61 | 61 | 58 | | 58 |
| Mean ± SD | 125.0 ± 10.4 | 81.8 ± 7.9 | 123.3 ± 9.9 | 79.6 ± 7.4 | 126.8 ± 10.7 | | 84.1 ± 7.9 |
| Change from baseline | −11.7 ± 10.8*** | −6.4 ± 6.7*** | −14.1 ± 12.5*** | −8.3 ± 7.5*** | −9.3 ± 8.2*** | | −4.3 ± 5.0*** |
| Bedtime home BP |  |  |  |  |  | |  |
| Baseline, *n* | 121 | 121 | 62 | 62 | 59 | | 59 |
| Mean ± SD | 131.0 ± 13.4 | 82.3 ± 8.7 | 131.7 ± 15.0 | 82.0 ± 8.8 | 130.3 ± 11.7 | | 82.7 ± 8.6 |
| Week 2, *n* | 119 | 119 | 61 | 61 | 58 | | 58 |
| Mean ± SD | 124.6 ± 12.2 | 79.6 ± 8.6 | 122.1 ± 12.4 | 76.9 ± 7.5 | 127.3 ± 11.4 | | 82.4 ± 8.8 |
| Change from baseline | −6.6 ± 9.0 | −2.8 ± 5.8 | −10.0 ± 9.5 | −5.1 ± 5.8 | −3.1 ± 6.9 | | −0.3 ± 4.6 |
| Week 4, *n* | 117 | 117 | 60 | 60 | 57 | | 57 |
| Mean ± SD | 122.7 ± 12.8 | 78.1 ± 8.6 | 120.8 ± 13.8 | 75.7 ± 8.3 | 124.7 ± 11.4 | | 80.6 ± 8.4 |
| Change from baseline | −8.6 ± 9.3 | −4.4 ± 5.7 | −11.3 ± 10.4 | −6.3 ± 6.2 | −5.8 ± 7.0 | | −2.4 ± 4.4 |
| Week 12, *n* | 112 | 112 | 58 | 58 | 54 | | 54 |
| Mean ± SD | 120.7 ± 11.3 | 76.5 ± 8.8 | 117.8 ± 11.5 | 73.5 ± 8.5 | 123.7 ± 10.4 | | 79.7 ± 8.0 |
| Change from baseline | −10.7 ± 10.4*** | −6.0 ± 6.7*** | −14.2 ± 11.5*** | −8.4 ± 7.0*** | −6.9 ± 7.6*** | | −3.4 ± 5.4*** |
| EOT, *n* | 119 | 119 | 61 | 61 | 58 | | 58 |
| Mean ± SD | 120.5 ± 11.2 | 76.6 ± 8.6 | 117.6 ± 11.5 | 73.8 ± 8.5 | 123.6 ± 10.1 | | 79.7 ± 7.7 |
| Change from baseline | −10.7 ± 10.4*** | −5.7 ± 6.8*** | −14.5 ± 11.4*** | −8.3 ± 7.0*** | −6.7 ± 7.5*** | | −3.1 ± 5.5*** |
| Office BP |  |  |  |  |  | |  |
| Baseline, *n* | 121 | 121 | 62 | 62 | 59 | | 59 |
| Mean ± SD | 141.6 ± 15.3 | 86.6 ± 10.9 | 141.5 ± 17.3 | 84.4 ± 12.1 | 141.8 ± 13.1 | | 88.9 ± 9.1 |
| Week 4, *n* | 117 | 117 | 60 | 60 | 57 | | 57 |
| Mean ± SD | 130.8 ± 14.0 | 81.0 ± 11.6 | 129.9 ± 15.1 | 78.7 ± 12.5 | 131.7 ± 12.8 | | 83.4 ± 10.1 |
| Change from baseline | −10.8 ± 13.8 | −5.6 ± 8.9 | −12.0 ± 15.9 | −5.8 ± 9.6 | −9.5 ± 11.0 | | −5.5 ± 8.2 |
| Week 12, *n* | 112 | 112 | 58 | 58 | 54 | | 54 |
| Mean ± SD | 129.4 ± 12.2 | 78.9 ± 11.3 | 128.1 ± 13.3 | 75.7 ± 10.9 | 130.7 ± 10.9 | | 82.3 ± 10.9 |
| Change from baseline | −12.2 ± 13.6*** | −7.9 ± 8.9*** | −14.0 ± 16.0*** | −8.8 ± 9.5*** | −10.3 ± 10.3*** | | −6.8 ± 8.2*** |
| EOT, *n* | 119 | 119 | 61 | 61 | 58 | | 58 |
| Mean ± SD | 129.4 ± 12.4 | 78.8 ± 11.3 | 127.7 ± 13.1 | 75.4 ± 11.0 | 131.2 ± 11.4 | | 82.3 ± 10.7 |
| Change from baseline | −12.1 ± 13.6*** | −7.9 ± 8.8*** | −14.0 ± 15.7*** | −9.1 ± 9.3*** | −10.0 ± 10.7*** | | −6.6 ± 8.0*** |

****p* <0.001 vs baseline, paired *t*-test.

*p*-values were not calculated for changes from baseline to Week 4.

*ARB* angiotensin receptor blocker, *BP* blood pressure, *CCB* calcium channel blocker, *DBP* diastolic blood pressure, *EOT* end of treatment, *SBP* systolic blood pressure.

**Table S4.** Achievement rate of target BP levels at Week 12 (full analysis set)

| **BP** | **Total**  ***N* = 116** | **ARB** **subcohort**  ***n* = 62** | **CCB subcohort**  ***n* = 54** |
| --- | --- | --- | --- |
| Morning home BP |  |  |  |
| *n* (%)  95% CI | 14 (12.1)  [6.8, 19.4] | 10 (16.1)  [8.0, 27.7] | 4 (7.4)  [2.1, 17.9] |
| Bedtime home BP |  |  |  |
| *n* (%)  95% CI | 39 (33.6)  [25.1, 43.0] | 28 (45.2)  [32.5, 58.3] | 11 (20.4)  [10.6, 33.5] |
| Office BP |  |  |  |
| *n* (%)  95% CI | 41 (35.3)  [26.7, 44.8] | 27 (43.5)  [31.0, 56.7] | 14 (25.9)  [15.0, 39.7] |

95% CIs were calculated using the Clopper–Pearson method.

The achievement rate of target BP levels was defined as a home BP <125/75 mmHg and office BP <130/80 mmHg.

*ARB* angiotensin receptor blocker, *BP* blood pressure, *CCB* calcium channel blocker, *CI* confidence interval.

**Table S5.** Achievement rate of target BP levels at Week 12 (per protocol set)

| **BP** | **Total**  ***N* = 112** | **ARB** **subcohort**  ***n* = 58** | **CCB subcohort**  ***n* = 54** |
| --- | --- | --- | --- |
| Morning home BP |  |  |  |
| *n* (%)  95% CI | 14 (12.5)  [7.0, 20.1] | 10 (17.2)  [8.6, 29.4] | 4 (7.4)  [2.1, 17.9] |
| Bedtime home BP |  |  |  |
| *n* (%)  95% CI | 37 (33.0)  [24.4, 42.6] | 26 (44.8)  [31.7, 58.5] | 11 (20.4)  [10.6, 33.5] |
| Office BP |  |  |  |
| *n* (%)  95% CI | 39 (34.8)  [26.1, 44.4] | 25 (43.1)  [30.2, 56.8] | 14 (25.9)  [15.0, 39.7] |

95% CIs were calculated using the Clopper–Pearson method.

The achievement rate of target BP levels was defined as a home BP <125/75 mmHg and office BP <130/80 mmHg.

*ARB* angiotensin receptor blocker, *BP* blood pressure, *CCB* calcium channel blocker, *CI* confidence interval.

**Table S6.** Correlation of change in BP from baseline to Week 12 with urinary Na/K ratio and estimated 24-h urinary Na excretion at baseline (full analysis set)

| **Variables** |  | **Total**  ***N* = 126** | | | | **ARB subcohort**  ***n* = 67** | | | | **CCB subcohort**  ***n* = 59** | | | |
| --- | --- | --- | --- | --- | --- | --- | --- | --- | --- | --- | --- | --- | --- |
|  |  | **Pearson coefficient** | **Estimate** | **SE** | ***p*-value** | **Pearson coefficient** | **Estimate** | **SE** | ***p*-value** | **Pearson coefficient** | **Estimate** | **SE** | ***p*-value** |
| Baseline urinary Na/K ratio | | | | | | | | | | | | | |
| Morning home | SBP | 0.11 | 0.02 | 0.02 | 0.240 | 0.12 | 0.02 | 0.02 | 0.368 | 0.14 | 0.03 | 0.03 | 0.325 |
|  | DBP | 0.01 | 0.00 | 0.03 | 0.926 | 0.03 | 0.01 | 0.04 | 0.842 | 0.02 | 0.01 | 0.05 | 0.902 |
| Bedtime home | SBP | 0.02 | 0.00 | 0.02 | 0.835 | 0.05 | 0.01 | 0.02 | 0.680 | 0.00 | 0.00 | 0.03 | 0.980 |
|  | DBP | 0.02 | 0.01 | 0.03 | 0.818 | 0.03 | 0.01 | 0.04 | 0.841 | 0.07 | 0.02 | 0.04 | 0.630 |
| Office | SBP | −0.15 | −0.02 | 0.01 | 0.110 | −0.23 | −0.03 | 0.02 | 0.077 | 0.03 | 0.01 | 0.02 | 0.814 |
|  | DBP | −0.13 | −0.03 | 0.02 | 0.171 | −0.16 | −0.04 | 0.03 | 0.206 | −0.06 | −0.01 | 0.03 | 0.662 |
| Estimated 24-h urinary Na excretion^a^ | | | | | | | | | | | | | |
| Morning home | SBP | 0.09 | 0.34 | 0.37 | 0.356 | 0.09 | 0.28 | 0.43 | 0.508 | 0.12 | 0.66 | 0.78 | 0.401 |
|  | DBP | 0.03 | 0.19 | 0.61 | 0.759 | 0.06 | 0.36 | 0.72 | 0.618 | 0.00 | 0.01 | 1.30 | 0.992 |
| Bedtime home | SBP | 0.05 | 0.23 | 0.40 | 0.558 | 0.06 | 0.24 | 0.48 | 0.624 | 0.08 | 0.50 | 0.84 | 0.557 |
|  | DBP | 0.05 | 0.36 | 0.62 | 0.564 | 0.06 | 0.39 | 0.79 | 0.628 | 0.08 | 0.68 | 1.19 | 0.569 |
| Office | SBP | −0.18 | −0.57 | 0.29 | 0.057 | −0.25 | −0.67 | 0.33 | 0.048 | −0.07 | −0.30 | 0.62 | 0.630 |
|  | DBP | −0.09 | −0.46 | 0.46 | 0.321 | −0.12 | −0.55 | 0.57 | 0.340 | −0.05 | −0.30 | 0.78 | 0.704 |

^a^ This was a *post hoc* analysis.

*ARB* angiotensin receptor blocker, *BP* blood pressure, *CCB* calcium channel blocker, *DBP* diastolic blood pressure, *K* potassium, *Na* sodium, *SBP* systolic blood pressure, *SE* standard error.

就寝前血圧

**Table S7.** Correlation of change in BP from baseline to Week 12 with urinary Na/K ratio and estimated 24-h urinary Na excretion at baseline (per protocol set)^a^

| **Variables** |  | **Total**  ***N* = 121** | | | | **ARB subcohort**  ***n* = 62** | | | | **CCB subcohort**  ***n* = 59** | | | |
| --- | --- | --- | --- | --- | --- | --- | --- | --- | --- | --- | --- | --- | --- |
|  |  | **Pearson coefficient** | **Estimate** | **SE** | ***p*-value** | **Pearson coefficient** | **Estimate** | **SE** | ***p*-value** | **Pearson coefficient** | **Estimate** | **SE** | ***p*-value** |
| Baseline urinary Na/K ratio | | | | | | | | | | | | | |
| Morning home | SBP | 0.10 | 0.02 | 0.02 | 0.275 | 0.10 | 0.02 | 0.02 | 0.440 | 0.14 | 0.03 | 0.03 | 0.325 |
|  | DBP | −0.01 | 0.00 | 0.03 | 0.917 | −0.01 | 0.00 | 0.04 | 0.956 | 0.02 | 0.01 | 0.05 | 0.902 |
| Bedtime home | SBP | 0.01 | 0.00 | 0.02 | 0.938 | 0.03 | 0.01 | 0.02 | 0.823 | 0.00 | 0.00 | 0.03 | 0.980 |
|  | DBP | 0.01 | 0.00 | 0.03 | 0.885 | 0.01 | 0.00 | 0.04 | 0.955 | 0.07 | 0.02 | 0.04 | 0.630 |
| Office | SBP | −0.13 | −0.02 | 0.01 | 0.172 | −0.20 | −0.03 | 0.02 | 0.125 | 0.03 | 0.01 | 0.02 | 0.814 |
|  | DBP | −0.12 | −0.02 | 0.02 | 0.225 | −0.15 | −0.03 | 0.03 | 0.275 | −0.06 | −0.01 | 0.03 | 0.662 |
| Estimated 24-h urinary Na excretion | | | | | | | | | | | | | |
| Morning home | SBP | 0.08 | 0.31 | 0.38 | 0.427 | 0.06 | 0.21 | 0.44 | 0.638 | 0.12 | 0.66 | 0.78 | 0.401 |
|  | DBP | 0.00 | 0.03 | 0.63 | 0.961 | 0.02 | 0.11 | 0.76 | 0.888 | 0.00 | 0.01 | 1.30 | 0.992 |
| Bedtime home | SBP | 0.04 | 0.17 | 0.40 | 0.681 | 0.03 | 0.11 | 0.49 | 0.820 | 0.08 | 0.50 | 0.84 | 0.557 |
|  | DBP | 0.04 | 0.29 | 0.62 | 0.638 | 0.04 | 0.24 | 0.80 | 0.761 | 0.08 | 0.68 | 1.19 | 0.569 |
| Office | SBP | −0.15 | −0.49 | 0.30 | 0.108 | −0.22 | −0.56 | 0.34 | 0.104 | −0.07 | −0.30 | 0.62 | 0.630 |
|  | DBP | −0.07 | −0.37 | 0.47 | 0.434 | −0.09 | −0.40 | 0.59 | 0.495 | −0.05 | −0.30 | 0.78 | 0.704 |

^a^ This was a *post hoc* analysis.

*ARB* angiotensin receptor blocker, *BP* blood pressure, *CCB* calcium channel blocker, *DBP* diastolic blood pressure, *K* potassium, *Na* sodium, *SBP* systolic blood pressure, *SE* standard error.

就寝前血圧

**Table S8.** BP according to baseline urinary Na/K ratio (full analysis set)

|  | **Total**  ***N* = 126** | | | **ARB subcohort**  ***n* = 67** | | | **CCB subcohort**  ***n* = 59** | | |
| --- | --- | --- | --- | --- | --- | --- | --- | --- | --- |
| **Urinary Na/K ratio** | **Lower tertile** | **Middle tertile** | **Higher tertile** | **Lower tertile** | **Middle tertile** | **Higher tertile** | **Lower tertile** | **Middle tertile** | **Higher tertile** |
| Morning home SBP, mmHg |  |  |  |  |  |  |  |  |  |
| Baseline, *n* | 41 | 44 | 41 | 21 | 25 | 21 | 20 | 19 | 20 |
| Mean ± SD | 135.4 ± 14.2 | 137.4 ± 12.1 | 137.2 ± 9.8 | 136.8 ± 14.2 | 138.6 ± 13.0 | 136.1 ± 10.2 | 134.0 ± 14.4 | 135.8 ± 10.9 | 138.4 ± 9.5 |
| Week 12, *n* | 38 | 40 | 38 | 18 | 23 | 21 | 20 | 17 | 17 |
| Mean ± SD | 122.6 ± 9.7 | 126.4 ± 9.4 | 125.6 ± 12.1 | 121.6 ± 8.6 | 124.7 ± 8.7 | 123.1 ± 11.9 | 123.6 ± 10.7 | 128.6 ± 10.1 | 128.6 ± 12.0 |
| Change from baseline | −12.9 ± 12.5 | −12.1 ± 10.8 | −11.8 ± 9.6 | −15.6 ± 15.1 | −14.7 ± 12.6 | −13.0 ± 10.5 | −10.5 ± 9.3 | −8.5 ± 6.5 | −10.4 ± 8.6 |
| 95% CI | −17.0, −8.8 | −15.5, −8.6 | −15.0, −8.7 | −23.1, −8.1 | −20.2, −9.3 | −17.7, −8.2 | −14.8, −6.1 | −11.8, −5.1 | −14.8, −6.0 |
| *p*-value | <0.001 | <0.001 | <0.001 | <0.001 | <0.001 | <0.001 | <0.001 | <0.001 | <0.001 |
| EOT, *n* | 41 | 42 | 40 | 21 | 23 | 21 | 20 | 19 | 19 |
| Mean ± SD | 122.7 ± 9.8 | 126.3 ± 9.2 | 125.9 ± 11.9 | 121.9 ± 9.1 | 124.7 ± 8.7 | 123.1 ± 11.9 | 123.6 ± 10.7 | 128.3 ± 9.6 | 128.8 ± 11.5 |
| Change from baseline | −12.8 ± 12.4 | −11.5 ± 10.9 | −11.4 ± 9.5 | −15.0 ± 14.6 | −14.7 ± 12.6 | −13.0 ± 10.5 | −10.5 ± 9.3 | −7.6 ± 6.8 | −9.7 ± 8.4 |
| 95% CI | −16.7, −8.9 | −14.9, −8.1 | −14.5, −8.4 | −21.6, −8.3 | −20.2, −9.3 | −17.7, −8.2 | −14.8, −6.1 | −10.9, −4.3 | −13.8, −5.7 |
| *p*-value | <0.001 | <0.001 | <0.001 | <0.001 | <0.001 | <0.001 | <0.001 | <0.001 | <0.001 |
| Morning home DBP, mmHg |  |  |  |  |  |  |  |  |  |
| Baseline, *n* | 41 | 44 | 41 | 21 | 25 | 21 | 20 | 19 | 20 |
| Mean ± SD | 87.6 ± 9.8 | 87.5 ± 9.3 | 88.8 ± 7.7 | 88.0 ± 9.8 | 86.8 ± 9.4 | 88.3 ± 8.6 | 87.1 ± 10.1 | 88.5 ± 9.4 | 89.4 ± 6.9 |
| Week 12, *n* | 38 | 40 | 38 | 18 | 23 | 21 | 20 | 17 | 17 |
| Mean ± SD | 81.2 ± 6.6 | 81.7 ± 8.6 | 82.3 ± 8.6 | 79.4 ± 6.5 | 79.0 ± 6.1 | 80.2 ± 9.1 | 82.8 ± 6.4 | 85.2 ± 10.3 | 84.9 ± 7.5 |
| Change from baseline | −6.5 ± 7.4 | −6.6 ± 7.4 | −7.0 ± 4.9 | −8.9 ± 8.5 | −8.6 ± 8.6 | −8.0 ± 5.1 | −4.4 ± 5.7 | −3.8 ± 4.4 | −5.6 ± 4.5 |
| 95% CI | −8.9, −4.1 | −8.9, −4.2 | −8.6, −5.4 | −13.1, −4.7 | −12.3, −4.8 | −10.4, −5.7 | −7.0, −1.7 | −6.1, −1.6 | −8.0, −3.3 |
| *p*-value | <0.001 | <0.001 | <0.001 | <0.001 | <0.001 | <0.001 | 0.003 | 0.003 | <0.001 |
| EOT, *n* | 41 | 42 | 40 | 21 | 23 | 21 | 20 | 19 | 19 |
| Mean ± SD | 81.3 ± 6.5 | 81.6 ± 8.4 | 82.5 ± 8.5 | 80.0 ± 6.5 | 79.0 ± 6.1 | 80.2 ± 9.1 | 82.8 ± 6.4 | 84.8 ± 9.8 | 84.9 ± 7.4 |
| Change from baseline | −6.2 ± 7.6 | −6.4 ± 7.5 | −6.6 ± 5.1 | −8.0 ± 8.7 | −8.6 ± 8.6 | −8.0 ± 5.1 | −4.4 ± 5.7 | −3.7 ± 4.7 | −5.0 ± 4.8 |
| 95% CI | −8.6, −3.9 | −8.7, −4.0 | −8.2, −5.0 | −12.0, −4.1 | −12.3, −4.8 | −10.4, −5.7 | −7.0, −1.7 | −6.0, −1.4 | −7.3, −2.7 |
| *p*-value | <0.001 | <0.001 | <0.001 | <0.001 | <0.001 | <0.001 | 0.003 | 0.003 | <0.001 |
| Bedtime home SBP, mmHg |  |  |  |  |  |  |  |  |  |
| Baseline, *n* | 41 | 44 | 41 | 21 | 25 | 21 | 20 | 19 | 20 |
| Mean ± SD | 132.5 ± 13.6 | 129.5 ± 13.2 | 129.6 ± 14.2 | 134.1 ± 16.2 | 129.6 ± 14.0 | 128.7 ± 15.8 | 130.9 ± 10.4 | 129.4 ± 12.5 | 130.5 ± 12.5 |
| Week 12, *n* | 38 | 40 | 38 | 18 | 23 | 21 | 20 | 17 | 17 |
| Mean ± SD | 120.7 ± 11.7 | 121.1 ± 10.9 | 119.1 ± 11.7 | 117.6 ± 12.9 | 119.1 ± 10.4 | 115.3 ± 11.5 | 123.5 ± 10.0 | 123.8 ± 11.3 | 123.8 ± 10.5 |
| Change from baseline | −11.8 ± 12.4 | −9.7 ± 8.8 | −10.4 ± 9.5 | −16.7 ± 15.2 | −12.1 ± 7.8 | −13.4 ± 10.5 | −7.4 ± 7.0 | −6.5 ± 9.2 | −6.8 ± 6.7 |
| 95% CI | −15.9, −7.7 | −12.5, −6.9 | −13.6, −7.3 | −24.3, −9.2 | −15.5, −8.8 | −18.2, −8.6 | −10.7, −4.1 | −11.2, −1.7 | −10.3, −3.4 |
| *p*-value | <0.001 | <0.001 | <0.001 | <0.001 | <0.001 | <0.001 | <0.001 | 0.011 | <0.001 |
| EOT, *n* | 41 | 42 | 40 | 21 | 23 | 21 | 20 | 19 | 19 |
| Mean ± SD | 120.1 ± 11.8 | 121.1 ± 10.7 | 119.3 ± 11.5 | 117.0 ± 12.8 | 119.1 ± 10.4 | 115.3 ± 11.5 | 123.5 ± 10.0 | 123.5 ± 10.7 | 123.7 ± 10.0 |
| Change from baseline | −12.4 ± 12.4 | −9.3 ± 8.8 | −10.3 ± 9.4 | −17.1 ± 14.5 | −12.1 ± 7.8 | −13.4 ± 10.5 | −7.4 ± 7.0 | −5.9 ± 9.0 | −6.9 ± 6.6 |
| 95% CI | −16.3, −8.5 | −12.1, −6.6 | −13.3, −7.3 | −23.8, −10.5 | −15.5, −8.8 | −18.2, −8.6 | −10.7, −4.1 | −10.2, −1.6 | −10.1, −3.7 |
| *p*-value | <0.001 | <0.001 | <0.001 | <0.001 | <0.001 | <0.001 | <0.001 | 0.010 | <0.001 |
| Bedtime home DBP, mmHg |  |  |  |  |  |  |  |  |  |
| Baseline, *n* | 41 | 44 | 41 | 21 | 25 | 21 | 20 | 19 | 20 |
| Mean ± SD | 83.4 ± 8.5 | 80.8 ± 9.8 | 81.8 ± 8.9 | 83.6 ± 9.4 | 79.2 ± 8.8 | 81.7 ± 10.5 | 83.2 ± 7.8 | 82.8 ± 11.0 | 82.0 ± 7.1 |
| Week 12, *n* | 38 | 40 | 38 | 18 | 23 | 21 | 20 | 17 | 17 |
| Mean ± SD | 76.6 ± 8.7 | 76.4 ± 8.8 | 76.2 ± 9.4 | 73.7 ± 8.4 | 73.5 ± 7.3 | 73.5 ± 10.6 | 79.3 ± 8.2 | 80.2 ± 9.3 | 79.6 ± 6.6 |
| Change from baseline | −6.7 ± 7.9 | −5.3 ± 5.9 | −5.9 ± 5.8 | −9.9 ± 9.3 | −6.9 ± 5.0 | −8.2 ± 5.9 | −3.9 ± 5.2 | −3.2 ± 6.5 | −3.2 ± 4.6 |
| 95% CI | −9.3, −4.1 | −7.2, −3.4 | −7.9, −4.0 | −14.5, −5.3 | −9.0, −4.7 | −10.9, −5.5 | −6.3, −1.5 | −6.5, 0.2 | −5.5, −0.8 |
| *p*-value | <0.001 | <0.001 | <0.001 | <0.001 | <0.001 | <0.001 | 0.003 | 0.060 | 0.011 |
| EOT, *n* | 41 | 42 | 40 | 21 | 23 | 21 | 20 | 19 | 19 |
| Mean ± SD | 76.7 ± 8.5 | 76.5 ± 8.6 | 76.4 ± 9.2 | 74.3 ± 8.2 | 73.5 ± 7.3 | 73.5 ± 10.6 | 79.3 ± 8.2 | 80.1 ± 8.8 | 79.7 ± 6.2 |
| Change from baseline | −6.7 ± 7.9 | −5.0 ± 6.0 | −5.5 ± 6.2 | −9.3 ± 9.2 | −6.9 ± 5.0 | −8.2 ± 5.9 | −3.9 ± 5.2 | −2.7 ± 6.5 | −2.5 ± 5.1 |
| 95% CI | −9.1, −4.2 | −6.9, −3.1 | −7.5, −3.5 | −13.5, −5.1 | −9.0, −4.7 | −10.9, −5.5 | −6.3, −1.5 | −5.9, 0.4 | −5.0, −0.1 |
| *p*-value | <0.001 | <0.001 | <0.001 | <0.001 | <0.001 | <0.001 | 0.003 | 0.082 | 0.046 |
| Office SBP, mmHg |  |  |  |  |  |  |  |  |  |
| Baseline, *n* | 41 | 44 | 41 | 21 | 25 | 21 | 20 | 19 | 20 |
| Mean ± SD | 140.2 ± 14.3 | 138.9 ± 15.0 | 145.4 ± 15.8 | 142.4 ± 15.4 | 137.6 ± 18.1 | 144.0 ± 16.8 | 137.9 ± 12.9 | 140.6 ± 9.7 | 146.8 ± 15.0 |
| Week 12, *n* | 38 | 40 | 38 | 18 | 23 | 21 | 20 | 17 | 17 |
| Mean ± SD | 129.1 ± 11.3 | 128.2 ± 13.6 | 129.3 ± 13.1 | 129.2 ± 12.1 | 127.3 ± 15.5 | 125.2 ± 13.7 | 129.0 ± 10.9 | 129.2 ± 10.9 | 134.4 ± 10.7 |
| Change from baseline | −11.7 ± 14.6 | −10.4 ± 12.1 | −15.6 ± 14.1 | −14.7 ± 17.5 | −10.3 ± 14.6 | −18.9 ± 15.2 | −9.0 ± 11.2 | −10.5 ± 7.9 | −11.6 ± 11.7 |
| 95% CI | −16.5, −6.9 | −14.3, −6.5 | −20.2, −11.0 | −23.4, −6.0 | −16.6, −4.0 | −25.8, −11.9 | −14.2, −3.7 | −14.6, −6.5 | −17.6, −5.6 |
| *p*-value | <0.001 | <0.001 | <0.001 | 0.002 | 0.003 | <0.001 | 0.002 | <0.001 | 0.001 |
| EOT, *n* | 41 | 42 | 40 | 21 | 23 | 21 | 20 | 19 | 19 |
| Mean ± SD | 128.4 ± 11.2 | 128.8 ± 14.4 | 129.5 ± 12.8 | 127.9 ± 11.7 | 127.3 ± 15.5 | 125.2 ± 13.7 | 129.0 ± 10.9 | 130.6 ± 13.0 | 134.3 ± 10.1 |
| Change from baseline | −11.8 ± 14.3 | −10.2 ± 12.6 | −15.2 ± 13.8 | −14.5 ± 16.5 | −10.3 ± 14.6 | −18.9 ± 15.2 | −9.0 ± 11.2 | −10.0 ± 10.0 | −11.1 ± 11.2 |
| 95% CI | −16.3, −7.3 | −14.1, −6.2 | −19.6, −10.7 | −22.0, −6.9 | −16.6, −4.0 | −25.8, −11.9 | −14.2, −3.7 | −14.8, −5.2 | −16.5, −5.7 |
| *p*-value | <0.001 | <0.001 | <0.001 | 0.001 | 0.003 | <0.001 | 0.002 | <0.001 | <0.001 |
| Office DBP, mmHg |  |  |  |  |  |  |  |  |  |
| Baseline, *n* | 41 | 44 | 41 | 21 | 25 | 21 | 20 | 19 | 20 |
| Mean ± SD | 86.7 ± 11.7 | 83.1 ± 9.3 | 89.9 ± 10.4 | 85.5 ± 13.8 | 80.8 ± 8.5 | 87.5 ± 12.1 | 88.0 ± 9.1 | 86.2 ± 9.7 | 92.4 ± 7.8 |
| Week 12, n | 38 | 40 | 38 | 18 | 23 | 21 | 20 | 17 | 17 |
| Mean ± SD | 78.7 ± 9.8 | 78.2 ± 12.7 | 79.6 ± 11.3 | 75.8 ± 12.1 | 75.7 ± 9.9 | 75.9 ± 11.2 | 81.3 ± 6.5 | 81.6 ± 15.5 | 84.2 ± 9.9 |
| Change from baseline | −8.4 ± 7.2 | −5.1 ± 9.8 | −10.3 ± 8.7 | −10.3 ± 8.2 | −5.1 ± 9.5 | −11.7 ± 9.1 | −6.7 ± 5.8 | −5.1 ± 10.4 | −8.6 ± 8.2 |
| 95% CI | −10.8, −6.1 | −8.2, −1.9 | −13.2, −7.4 | −14.4, −6.3 | −9.2, −1.0 | −15.8, −7.5 | −9.4, −4.0 | −10.4, 0.3 | −12.9, −4.4 |
| *p*-value | <0.001 | 0.002 | <0.001 | <0.001 | 0.018 | <0.001 | <0.001 | 0.062 | <0.001 |
| EOT, *n* | 41 | 42 | 40 | 21 | 23 | 21 | 20 | 19 | 19 |
| Mean ± SD | 77.9 ± 10.2 | 78.3 ± 12.6 | 80.0 ± 11.2 | 74.7 ± 12.0 | 75.7 ± 9.9 | 75.9 ± 11.2 | 81.3 ± 6.5 | 81.4 ± 14.9 | 84.5 ± 9.4 |
| Change from baseline | −8.8 ± 7.1 | −5.0 ± 9.6 | −10.1 ± 8.6 | −10.9 ± 7.7 | −5.1 ± 9.5 | −11.7 ± 9.1 | −6.7 ± 5.8 | −4.8 ± 10.0 | −8.3 ± 7.9 |
| 95% CI | −11.1, −6.6 | −8.0, −2.0 | −12.8, −7.3 | −14.4, −7.3 | −9.2, −1.0 | −15.8, −7.5 | −9.4, −4.0 | −9.7, 0.0 | −12.0, −4.5 |
| *p*-value | <0.001 | 0.002 | <0.001 | <0.001 | 0.018 | <0.001 | <0.001 | 0.050 | <0.001 |

*ARB* angiotensin receptor blocker, *BP* blood pressure, *CCB* calcium channel blocker, *CI* confidence interval, *DBP* diastolic blood pressure, *EOT* end of treatment, *K* potassium, *Na* sodium, *SBP* systolic blood pressure.

**Table S9.** BP according to baseline estimated 24-h urinary Na excretion (full analysis set)^a^

|  | **Total**  ***N* = 126** | | | **ARB subcohort**  ***n* = 67** | | | **CCB subcohort**  ***n* = 59** | | |
| --- | --- | --- | --- | --- | --- | --- | --- | --- | --- |
| **Estimated 24-h urinary Na excretion** | **Lower tertile**^b^ | **Middle tertile**^c^ | **Higher tertile**^d^ | **Lower tertile**^b^ | **Middle tertile**^c^ | **Higher tertile**^d^ | **Lower tertile**^b^ | **Middle tertile**^c^ | **Higher tertile**^d^ |
| Morning home SBP, mmHg |  |  |  |  |  |  |  |  |  |
| Baseline, *n* | 41 | 44 | 41 | 23 | 25 | 19 | 18 | 19 | 22 |
| Mean ± SD | 134.8 ± 14.1 | 138.6 ± 11.5 | 136.7 ± 10.4 | 134.8 ± 12.8 | 140.0 ± 12.9 | 136.7 ± 11.3 | 134.7 ± 16.0 | 136.7 ± 9.3 | 136.6 ± 9.8 |
| Week 12, *n* | 38 | 42 | 36 | 20 | 23 | 19 | 18 | 19 | 17 |
| Mean ± SD | 122.8 ± 11.4 | 125.7 ± 9.6 | 126.1 ± 10.5 | 121.4 ± 9.1 | 124.2 ± 11.0 | 124.2 ± 9.2 | 124.5 ± 13.5 | 127.6 ± 7.4 | 128.2 ± 11.7 |
| Change from baseline | −12.0 ± 12.0 | −13.1 ± 12.0 | −11.4 ± 8.4 | −13.7 ± 13.6 | −16.5 ± 14.5 | −12.5 ± 8.6 | −10.2 ± 10.1 | −9.1 ± 6.2 | −10.2 ± 8.3 |
| 95% CI | −16.0, −8.1 | −16.9, −9.4 | −14.3, −8.6 | −20.0, −7.4 | −22.8, −10.2 | −16.7, −8.4 | −15.2, −5.2 | −12.1, −6.1 | −14.5, −6.0 |
| *p*-value | <0.001 | <0.001 | <0.001 | <0.001 | <0.001 | <0.001 | <0.001 | <0.001 | <0.001 |
| EOT, *n* | 40 | 43 | 40 | 22 | 24 | 19 | 18 | 19 | 21 |
| Mean ± SD | 123.2 ± 11.3 | 125.4 ± 9.7 | 126.2 ± 10.1 | 122.1 ± 9.4 | 123.6 ± 11.1 | 124.2 ± 9.2 | 124.5 ± 13.5 | 127.6 ± 7.4 | 128.1 ± 10.7 |
| Change from baseline | −11.7 ± 11.9 | −13.4 ± 12.0 | −10.5 ± 8.6 | −12.9 ± 13.3 | −16.8 ± 14.3 | −12.5 ± 8.6 | −10.2 ± 10.1 | −9.1 ± 6.2 | −8.7 ± 8.4 |
| 95% CI | −15.5, −7.8 | −17.1, −9.7 | −13.2, −7.8 | −18.8, −7.0 | −22.9, −10.8 | −16.7, −8.4 | −15.2, −5.2 | −12.1, −6.1 | −12.5, −4.9 |
| *p*-value | <0.001 | <0.001 | <0.001 | <0.001 | <0.001 | <0.001 | <0.001 | <0.001 | <0.001 |
| Morning home DBP, mmHg |  |  |  |  |  |  |  |  |  |
| Baseline, *n* | 41 | 44 | 41 | 23 | 25 | 19 | 18 | 19 | 22 |
| Mean ± SD | 86.2 ± 10.1 | 90.6 ± 9.2 | 86.9 ± 6.8 | 84.5 ± 9.1 | 92.1 ± 9.7 | 85.6 ± 6.2 | 88.3 ± 11.3 | 88.6 ± 8.3 | 88.0 ± 7.2 |
| Week 12, *n* | 38 | 42 | 36 | 20 | 23 | 19 | 18 | 19 | 17 |
| Mean ± SD | 80.8 ± 8.8 | 83.0 ± 7.7 | 81.1 ± 7.2 | 77.9 ± 8.5 | 82.1 ± 6.8 | 78.3 ± 5.6 | 84.1 ± 8.2 | 84.2 ± 8.7 | 84.3 ± 7.7 |
| Change from baseline | −6.0 ± 6.4 | −7.6 ± 8.5 | −6.3 ± 4.0 | −7.6 ± 6.5 | −10.2 ± 10.0 | −7.4 ± 3.7 | −4.2 ± 5.9 | −4.5 ± 4.8 | −5.1 ± 4.1 |
| 95% CI | −8.1, −3.9 | −10.2, −4.9 | −7.7, −4.9 | −10.6, −4.6 | −14.5, −5.8 | −9.2, −5.6 | −7.1, −1.3 | −6.8, −2.1 | −7.2, −3.0 |
| *p*-value | <0.001 | <0.001 | <0.001 | <0.001 | <0.001 | <0.001 | 0.007 | <0.001 | <0.001 |
| EOT, *n* | 40 | 43 | 40 | 22 | 24 | 19 | 18 | 19 | 21 |
| Mean ± SD | 81.1 ± 8.7 | 82.9 ± 7.7 | 81.3 ± 7.1 | 78.7 ± 8.5 | 81.8 ± 6.8 | 78.3 ± 5.6 | 84.1 ± 8.2 | 84.2 ± 8.7 | 84.1 ± 7.2 |
| Change from baseline | −5.6 ± 6.5 | −7.8 ± 8.5 | −5.8 ± 4.5 | −6.6 ± 6.9 | −10.4 ± 9.9 | −7.4 ± 3.7 | −4.2 ± 5.9 | −4.5 ± 4.8 | −4.3 ± 4.7 |
| 95% CI | −7.6, −3.5 | −10.4, −5.2 | −7.2, −4.3 | −9.7, −3.6 | −14.5, −6.2 | −9.2, −5.6 | −7.1, −1.3 | −6.8, −2.1 | −6.5, −2.2 |
| *p*-value | <0.001 | <0.001 | <0.001 | <0.001 | <0.001 | <0.001 | 0.007 | <0.001 | <0.001 |
| Bedtime home SBP, mmHg |  |  |  |  |  |  |  |  |  |
| Baseline, *n* | 41 | 44 | 41 | 23 | 25 | 19 | 18 | 19 | 22 |
| Mean ± SD | 131.2 ± 14.3 | 131.2 ± 13.5 | 129.1 ± 13.3 | 130.3 ± 15.5 | 132.4 ± 16.2 | 128.9 ± 14.3 | 132.3 ± 13.0 | 129.5 ± 9.2 | 129.3 ± 12.7 |
| Week 12, *n* | 38 | 42 | 36 | 20 | 23 | 19 | 18 | 19 | 17 |
| Mean ± SD | 120.4 ± 11.0 | 120.5 ± 12.3 | 120.0 ± 11.0 | 117.3 ± 10.3 | 117.9 ± 13.4 | 116.9 ± 10.5 | 123.8 ± 11.0 | 123.8 ± 10.1 | 123.4 ± 10.7 |
| Change from baseline | −11.3 ± 11.2 | −11.1 ± 11.4 | −9.4 ± 7.6 | −13.7 ± 13.1 | −15.6 ± 12.3 | −12.0 ± 7.3 | −8.6 ± 8.2 | −5.7 ± 7.5 | −6.6 ± 7.1 |
| 95% CI | −15.0, −7.6 | −14.7, −7.6 | −12.0, −6.9 | −19.8, −7.6 | −20.9, −10.3 | −15.5, −8.5 | −12.6, −4.5 | −9.3, −2.1 | −10.2, −2.9 |
| *p*-value | <0.001 | <0.001 | <0.001 | <0.001 | <0.001 | <0.001 | <0.001 | 0.004 | 0.002 |
| EOT, *n* | 40 | 43 | 40 | 22 | 24 | 19 | 18 | 19 | 21 |
| Mean ± SD | 120.3 ± 10.9 | 120.1 ± 12.5 | 120.2 ± 10.5 | 117.5 ± 10.2 | 117.1 ± 13.6 | 116.9 ± 10.5 | 123.8 ± 11.0 | 123.8 ± 10.1 | 123.2 ± 9.7 |
| Change from baseline | −11.5 ± 11.2 | −11.5 ± 11.6 | −8.9 ± 7.6 | −13.8 ± 12.8 | −16.2 ± 12.3 | −12.0 ± 7.3 | −8.6 ± 8.2 | −5.7 ± 7.5 | −6.1 ± 6.9 |
| 95% CI | −15.0, −7.9 | −15.1, −8.0 | −11.4, −6.5 | −19.5, −8.1 | −21.4, −11.0 | −15.5, −8.5 | −12.6, −4.5 | −9.3, −2.1 | −9.3, −3.0 |
| *p*-value | <0.001 | <0.001 | <0.001 | <0.001 | <0.001 | <0.001 | <0.001 | 0.004 | <0.001 |
| Bedtime home DBP, mmHg |  |  |  |  |  |  |  |  |  |
| Baseline, *n* | 41 | 44 | 41 | 23 | 25 | 19 | 18 | 19 | 22 |
| Mean ± SD | 82.1 ± 9.8 | 84.0 ± 8.9 | 79.6 ± 8.2 | 80.2 ± 9.8 | 84.6 ± 9.3 | 78.5 ± 8.9 | 84.5 ± 9.6 | 83.3 ± 8.5 | 80.6 ± 7.7 |
| Week 12, *n* | 38 | 42 | 36 | 20 | 23 | 19 | 18 | 19 | 17 |
| Mean ± SD | 76.3 ± 9.8 | 77.3 ± 8.3 | 75.4 ± 8.6 | 72.9 ± 9.5 | 75.6 ± 7.8 | 71.8 ± 8.9 | 80.1 ± 9.0 | 79.5 ± 8.6 | 79.5 ± 6.3 |
| Change from baseline | −6.4 ± 7.3 | −6.8 ± 7.4 | −4.5 ± 4.4 | −8.3 ± 8.0 | −9.3 ± 7.8 | −6.7 ± 3.4 | −4.4 ± 6.0 | −3.8 ± 5.8 | −2.0 ± 4.0 |
| 95% CI | −8.8, −4.1 | −9.1, −4.5 | −6.0, −3.0 | −12.0, −4.6 | −12.7, −5.9 | −8.4, −5.1 | −7.4, −1.4 | −6.6, −1.1 | −4.1, 0.1 |
| *p*-value | <0.001 | <0.001 | <0.001 | <0.001 | <0.001 | <0.001 | 0.006 | 0.010 | 0.056 |
| EOT, *n* | 40 | 43 | 40 | 22 | 24 | 19 | 18 | 19 | 21 |
| Mean ± SD | 76.6 ± 9.7 | 77.2 ± 8.3 | 75.9 ± 8.2 | 73.7 ± 9.4 | 75.3 ± 7.7 | 71.8 ± 8.9 | 80.1 ± 9.0 | 79.5 ± 8.6 | 79.5 ± 5.7 |
| Change from baseline | −6.2 ± 7.2 | −7.0 ± 7.4 | −3.9 ± 4.9 | −7.6 ± 7.9 | −9.6 ± 7.7 | −6.7 ± 3.4 | −4.4 ± 6.0 | −3.8 ± 5.8 | −1.2 ± 4.7 |
| 95% CI | −8.5, −3.8 | −9.3, −4.8 | −5.4, −2.3 | −11.1, −4.1 | −12.8, −6.3 | −8.4, −5.1 | −7.4, −1.4 | −6.6, −1.1 | −3.4, 0.9 |
| *p*-value | <0.001 | <0.001 | <0.001 | <0.001 | <0.001 | <0.001 | 0.006 | 0.010 | 0.237 |
| Office SBP, mmHg |  |  |  |  |  |  |  |  |  |
| Baseline, *n* | 41 | 44 | 41 | 23 | 25 | 19 | 18 | 19 | 22 |
| Mean ± SD | 138.1 ± 13.3 | 139.1 ± 13.0 | 147.2 ± 17.5 | 136.5 ± 13.7 | 139.4 ± 15.1 | 149.0 ± 20.3 | 140.2 ± 12.9 | 138.8 ± 10.2 | 145.7 ± 15.0 |
| Week 12, *n* | 38 | 42 | 36 | 20 | 23 | 19 | 18 | 19 | 17 |
| Mean ± SD | 127.2 ± 11.8 | 127.0 ± 11.6 | 132.6 ± 14.1 | 125.7 ± 12.3 | 124.9 ± 13.0 | 131.4 ± 15.9 | 128.9 ± 11.3 | 129.6 ± 9.4 | 133.9 ± 12.0 |
| Change from baseline | −10.8 ± 15.0 | −12.6 ± 11.4 | −14.2 ± 14.7 | −10.4 ± 18.3 | −15.5 ± 12.0 | −17.6 ± 17.2 | −11.3 ± 10.9 | −9.2 ± 9.9 | −10.5 ± 10.7 |
| 95% CI | −15.7, −5.8 | −16.2, −9.1 | −19.2, −9.2 | −18.9, −1.8 | −20.7, −10.3 | −25.9, −9.3 | −16.7, −5.8 | −13.9, −4.4 | −16.0, −5.0 |
| *p*-value | <0.001 | <0.001 | <0.001 | 0.020 | <0.001 | <0.001 | <0.001 | <0.001 | 0.001 |
| EOT, *n* | 40 | 43 | 40 | 22 | 24 | 19 | 18 | 19 | 21 |
| Mean ± SD | 126.8 ± 11.7 | 127.0 ± 11.5 | 133.2 ± 14.3 | 125.0 ± 11.9 | 124.8 ± 12.7 | 131.4 ± 15.9 | 128.9 ± 11.3 | 129.6 ± 9.4 | 134.7 ± 12.8 |
| Change from baseline | −11.1 ± 14.9 | −12.5 ± 11.3 | −13.4 ± 14.8 | −10.9 ± 17.8 | −15.1 ± 11.8 | −17.6 ± 17.2 | −11.3 ± 10.9 | −9.2 ± 9.9 | −9.7 ± 11.5 |
| 95% CI | −15.8, −6.3 | −16.0, −9.0 | −18.2, −8.7 | −18.8, −3.0 | −20.1, −10.1 | −25.9, −9.3 | −16.7, −5.8 | −13.9, −4.4 | −14.9, −4.4 |
| *p*-value | <0.001 | <0.001 | <0.001 | 0.009 | <0.001 | <0.001 | <0.001 | <0.001 | 0.001 |
| Office DBP, mmHg |  |  |  |  |  |  |  |  |  |
| Baseline, *n* | 41 | 44 | 41 | 23 | 25 | 19 | 18 | 19 | 22 |
| Mean ± SD | 84.6 ± 11.5 | 86.4 ± 10.3 | 88.4 ± 10.4 | 81.2 ± 12.1 | 85.6 ± 10.9 | 86.7 ± 11.9 | 89.0 ± 9.1 | 87.5 ± 9.5 | 90.0 ± 9.0 |
| Week 12, *n* | 38 | 42 | 36 | 20 | 23 | 19 | 18 | 19 | 17 |
| Mean ± SD | 77.2 ± 9.3 | 79.2 ± 12.8 | 80.1 ± 11.5 | 73.2 ± 9.9 | 75.9 ± 11.1 | 78.4 ± 11.4 | 81.6 ± 6.2 | 83.2 ± 13.9 | 82.1 ± 11.6 |
| Change from baseline | −7.6 ± 8.5 | −7.6 ± 9.4 | −8.6 ± 8.7 | −7.8 ± 10.7 | −10.2 ± 7.6 | −8.3 ± 10.0 | −7.4 ± 5.3 | −4.4 ± 10.6 | −8.8 ± 7.4 |
| 95% CI | −10.4, −4.8 | −10.5, −4.6 | −11.5, −5.6 | −12.8, −2.7 | −13.5, −6.9 | −13.1, −3.5 | −10.1, −4.8 | −9.5, 0.7 | −12.6, −5.0 |
| *p*-value | <0.001 | <0.001 | <0.001 | 0.004 | <0.001 | 0.002 | <0.001 | 0.089 | <0.001 |
| EOT, *n* | 40 | 43 | 40 | 22 | 24 | 19 | 18 | 19 | 21 |
| Mean ± SD | 76.8 ± 9.4 | 78.8 ± 12.9 | 80.4 ± 11.1 | 73.0 ± 10.0 | 75.4 ± 11.2 | 78.4 ± 11.4 | 81.6 ± 6.2 | 83.2 ± 13.9 | 82.3 ± 10.8 |
| Change from baseline | −7.8 ± 8.3 | −7.8 ± 9.5 | −8.1 ± 8.5 | −8.1 ± 10.2 | −10.5 ± 7.6 | −8.3 ± 10.0 | −7.4 ± 5.3 | −4.4 ± 10.6 | −7.9 ± 7.2 |
| 95% CI | −10.5, −5.2 | −10.7, −4.9 | −10.8, −5.4 | −12.7, −3.6 | −13.8, −7.3 | −13.1, −3.5 | −10.1, −4.8 | −9.5, 0.7 | −11.2, −4.6 |
| *p*-value | <0.001 | <0.001 | <0.001 | 0.001 | <0.001 | 0.002 | <0.001 | 0.089 | <0.001 |

^a^ This was a *post hoc* analysis.

^b^ Lower tertile, <142.5 mEq/day.

^c^ Middle tertile, 142.5 to 189.9 mEq/day.

^d^ Higher tertile, ≥189.9 mEq/day.

*ARB* angiotensin receptor blocker, *BP* blood pressure, *CCB* calcium channel blocker, *CI* confidence interval, *DBP* diastolic blood pressure, *EOT* end of treatment, *Na* sodium, *SBP* systolic blood pressure.

**Table S10.** BP according to baseline urinary Na/K ratio (per protocol set)^a^

|  | **Total**  ***N* = 121** | | | **ARB subcohort**  ***n* = 62** | | | **CCB subcohort**  ***n* = 59** | | |
| --- | --- | --- | --- | --- | --- | --- | --- | --- | --- |
| **Urinary Na/K ratio** | **Lower tertile** | **Middle tertile** | **Higher tertile** | **Lower tertile** | **Middle tertile** | **Higher tertile** | **Lower tertile** | **Middle tertile** | **Higher tertile** |
| Morning home SBP, mmHg |  |  |  |  |  |  |  |  |  |
| Baseline, *n* | 40 | 41 | 40 | 21 | 21 | 20 | 19 | 20 | 20 |
| Mean ± SD | 135.1 ± 14.1 | 137.8 ± 12.3 | 137.2 ± 10.0 | 136.8 ± 14.2 | 139.0 ± 13.4 | 136.0 ± 10.5 | 133.1 ± 14.2 | 136.6 ± 11.1 | 138.4 ± 9.5 |
| Week 12, *n* | 37 | 38 | 37 | 18 | 20 | 20 | 19 | 18 | 17 |
| Mean ± SD | 122.1 ± 9.4 | 127.3 ± 9.3 | 125.5 ± 12.3 | 121.6 ± 8.6 | 125.5 ±8.3 | 122.8 ± 12.1 | 122.7 ± 10.2 | 129.3 ± 10.2 | 128.6 ± 12.0 |
| Change from baseline | −12.9 ± 12.6 | −11.5 ± 10.2 | −11.9 ± 9.7 | −15.6 ± 15.1 | −14.1 ± 12.3 | −13.2 ± 10.7 | −10.4 ±9.5 | −8.6 ± 6.4 | −10.4 ± 8.6 |
| 95% CI | −17.1, −8.7 | −14.8, −8.1 | −15.1, −8.6 | −23.1, −8.1 | −19.8, −8.3 | −18.2, −8.1 | −15.0, −5.8 | −11.8, −5.4 | −14.8, −6.0 |
| *p*-value | <0.001 | <0.001 | <0.001 | <0.001 | <0.001 | <0.001 | <0.001 | <0.001 | <0.001 |
| EOT, *n* | 40 | 40 | 39 | 21 | 20 | 20 | 19 | 20 | 19 |
| Mean ± SD | 122.3 ± 9.5 | 127.2 ± 9.1 | 125.7 ± 12.1 | 121.9 ± 9.1 | 125.5 ± 8.3 | 122.8 ± 12.1 | 122.7 ± 10.2 | 128.9 ± 9.7 | 128.8 ± 11.5 |
| Change from baseline | −12.8 ± 12.5 | −10.9 ± 10.3 | −11.5 ±9.7 | −15.0 ± 14.6 | −14.1 ± 12.3 | −13.2 ± 10.7 | −10.4 ± 9.5 | −7.8 ± 6.7 | −9.7 ± 8.4 |
| 95% CI | −16.8, −8.8 | −14.2, −7.6 | −14.6, −8.4 | −21.6, −8.3 | −19.8, −8.3 | −18.2, −8.1 | −15.0, −5.8 | −10.9, −4.6 | −13.8, −5.7 |
| *p*-value | <0.001 | <0.001 | <0.001 | <0.001 | <0.001 | <0.001 | <0.001 | <0.001 | <0.001 |
| Morning home DBP, mmHg |  |  |  |  |  |  |  |  |  |
| Baseline, *n* | 40 | 41 | 40 | 21 | 21 | 20 | 19 | 20 | 20 |
| Mean ± SD | 87.3 ± 9.8 | 88.0 ± 9.1 | 89.0 ± 7.7 | 88.0 ± 9.8 | 87.0 ± 8.8 | 88.7 ± 8.6 | 86.4 ± 9.9 | 89.1 ± 9.5 | 89.4 ± 6.9 |
| Week 12, *n* | 37 | 38 | 37 | 18 | 20 | 20 | 19 | 18 | 17 |
| Mean ± SD | 80.9 ± 6.4 | 81.9 ± 8.9 | 82.4 ± 8.8 | 79.4 ± 6.5 | 78.6 ± 6.3 | 80.2 ± 9.3 | 82.3 ± 6.2 | 85.6 ± 10.1 | 84.9 ± 7.5 |
| Change from baseline | −6.5 ± 7.5 | −6.3 ± 7.2 | −7.2 ± 4.9 | −8.9 ± 8.5 | −8.4 ± 8.6 | −8.5 ± 4.8 | −4.2 ± 5.8 | −4.1 ± 4.4 | −5.6 ± 4.5 |
| 95% CI | −9.0, −4.0 | −8.7, −4.0 | −8.8, −5.5 | −13.1, −4.7 | −12.4, −4.3 | −10.7, −6.2 | −7.0, −1.4 | −6.2, −1.9 | −8.0, −3.3 |
| *p*-value | <0.001 | <0.001 | <0.001 | <0.001 | <0.001 | <0.001 | 0.006 | 0.001 | <0.001 |
| EOT, *n* | 40 | 40 | 39 | 21 | 20 | 20 | 19 | 20 | 19 |
| Mean ± SD | 81.1 ± 6.4 | 81.9 ± 8.7 | 82.5 ± 8.7 | 80.0 ± 6.5 | 78.6 ± 6.3 | 80.2 ± 9.3 | 82.3 ± 6.2 | 85.2 ± 9.6 | 84.9 ± 7.4 |
| Change from baseline | −6.2 ± 7.7 | −6.1 ± 7.2 | −6.8 ± 5.1 | −8.0 ± 8.7 | −8.4 ± 8.6 | −8.5 ± 4.8 | −4.2 ± 5.8 | −3.9 ± 4.7 | −5.0 ± 4.8 |
| 95% CI | −8.6, −3.8 | −8.4, −3.8 | −8.4, −5.1 | −12.0, −4.1 | −12.4, −4.3 | −10.7, −6.2 | −7.0, −1.4 | −6.1, −1.7 | −7.3, −2.7 |
| *p*-value | <0.001 | <0.001 | <0.001 | <0.001 | <0.001 | <0.001 | 0.006 | 0.002 | <0.001 |
| Bedtime home SBP, mmHg |  |  |  |  |  |  |  |  |  |
| Baseline, *n* | 40 | 41 | 40 | 21 | 21 | 20 | 19 | 20 | 20 |
| Mean ± SD | 132.6 ± 13.8 | 130.2 ± 13.2 | 130.4 ± 13.5 | 134.1 ± 16.2 | 130.9 ± 14.4 | 130.2 ± 14.7 | 130.9 ± 10.7 | 129.5 ± 12.2 | 130.5 ± 12.5 |
| Week 12, *n* | 37 | 38 | 37 | 18 | 20 | 20 | 19 | 18 | 17 |
| Mean ± SD | 120.4 ± 11.7 | 121.8 ± 11.1 | 119.7 ± 11.3 | 117.6 ± 12.9 | 119.7 ± 10.9 | 116.2 ± 11.1 | 123.1 ± 10.2 | 124.1 ± 11.1 | 123.8 ± 10.5 |
| Change from baseline | −12.1 ± 12.4 | −9.3 ± 9.1 | −10.7 ± 9.5 | −16.7 ± 15.2 | −12.1 ± 8.3 | −14.0 ± 10.4 | −7.8 ± 7.0 | −6.1 ± 9.1 | −6.8 ± 6.7 |
| 95% CI | −16.3, −8.0 | −12.3, −6.3 | −13.9, −7.5 | −24.3, −9.2 | −16.0, −8.2 | −18.9, −9.1 | −11.2, −4.4 | −10.6, −1.6 | −10.3, −3.4 |
| *p*-value | <0.001 | <0.001 | <0.001 | <0.001 | <0.001 | <0.001 | <0.001 | 0.011 | <0.001 |
| EOT, *n* | 40 | 40 | 39 | 21 | 20 | 20 | 19 | 20 | 19 |
| Mean ± SD | 119.9 ± 11.9 | 121.8 ± 10.8 | 119.9 ± 11.1 | 117.0 ± 12.8 | 119.7 ± 10.9 | 116.2 ± 11.1 | 123.1 ± 10.2 | 123.9 ± 10.5 | 123.7 ± 10.0 |
| Change from baseline | −12.7 ± 12.4 | −8.9 ± 9.1 | −10.5 ± 9.4 | −17.1 ± 14.5 | −12.1 ± 8.3 | −14.0 ± 10.4 | −7.8 ± 7.0 | −5.6 ± 8.8 | −6.9 ± 6.6 |
| 95% CI | −16.7, −8.7 | −11.8, −5.9 | −13.6, −7.5 | −23.8, −10.5 | −16.0, −8.2 | −18.9, −9.1 | −11.2, −4.4 | −9.7, −1.5 | −10.1, −3.7 |
| *p*-value | <0.001 | <0.001 | <0.001 | <0.001 | <0.001 | <0.001 | <0.001 | 0.011 | <0.001 |
| Bedtime home DBP, mmHg |  |  |  |  |  |  |  |  |  |
| Baseline, *n* | 40 | 41 | 40 | 21 | 21 | 20 | 19 | 20 | 20 |
| Mean ± SD | 83.4 ± 8.6 | 81.2 ± 9.2 | 82.4 ± 8.2 | 83.6 ± 9.4 | 79.5 ± 7.3 | 82.8 ± 9.4 | 83.1 ± 8.0 | 83.0 ± 10.7 | 82.0 ± 7.1 |
| Week 12, *n* | 37 | 38 | 37 | 18 | 20 | 20 | 19 | 18 | 17 |
| Mean ± SD | 76.4 ± 8.7 | 76.3 ± 8.9 | 76.8 ± 9.0 | 73.7 ± 8.4 | 72.7 ± 7.1 | 74.3 ± 10.1 | 79.0 ± 8.3 | 80.4 ± 9.1 | 79.6 ± 6.6 |
| Change from baseline | −6.9 ± 7.9 | −5.1 ± 6.1 | −6.1 ± 5.9 | −9.9 ± 9.3 | −7.1 ± 5.4 | −8.5 ± 5.9 | −4.1 ± 5.2 | −3.0 ± 6.3 | −3.2 ± 4.6 |
| 95% CI | −9.6, −4.3 | −7.1, −3.1 | −8.0, −4.1 | −14.5, −5.3 | −9.6, −4.5 | −11.3, −5.7 | −6.6, −1.6 | −6.1, 0.1 | −5.5, −0.8 |
| *p*-value | <0.001 | <0.001 | <0.001 | <0.001 | <0.001 | <0.001 | 0.003 | 0.061 | 0.011 |
| EOT, *n* | 40 | 40 | 39 | 21 | 20 | 20 | 19 | 20 | 19 |
| Mean ± SD | 76.5 ± 8.5 | 76.5 ± 8.7 | 76.9 ± 8.8 | 74.3 ± 8.2 | 72.7 ± 7.1 | 74.3 ± 10.1 | 79.0 ± 8.3 | 80.4 ± 8.6 | 79.7 ± 6.2 |
| Change from baseline | −6.8 ± 7.9 | −4.8 ± 6.2 | −5.6 ± 6.2 | −9.3 ± 9.2 | −7.1 ± 5.4 | −8.5 ± 5.9 | −4.1 ± 5.2 | −2.6 ± 6.3 | −2.5 ± 5.1 |
| 95% CI | −9.4, −4.3 | −6.8, −2.8 | −7.6, −3.6 | −13.5, −5.1 | −9.6, −4.5 | −11.3, −5.7 | −6.6, −1.6 | −5.6, 0.4 | −5.0, −0.1 |
| *p*-value | <0.001 | <0.001 | <0.001 | <0.001 | <0.001 | <0.001 | 0.003 | 0.082 | 0.046 |
| Office SBP, mmHg |  |  |  |  |  |  |  |  |  |
| Baseline, *n* | 40 | 41 | 40 | 21 | 21 | 20 | 19 | 20 | 20 |
| Mean ± SD | 140.5 ± 14.3 | 138.9 ± 15.2 | 145.5 ± 16.0 | 142.4 ± 15.4 | 137.9 ± 19.2 | 144.2 ± 17.3 | 138.5 ± 13.0 | 140.0 ± 9.9 | 146.8 ± 15.0 |
| Week 12, *n* | 37 | 38 | 37 | 18 | 20 | 20 | 19 | 18 | 17 |
| Mean ± SD | 129.3 ± 11.4 | 128.8 ± 13.1 | 130.0 ± 12.5 | 129.2 ± 12.1 | 128.9 ± 15.1 | 126.4 ± 12.9 | 129.3 ± 11.1 | 128.8 ± 10.7 | 134.4 ± 10.7 |
| Change from baseline | −11.8 ± 14.8 | −9.9 ± 12.2 | −15.0 ± 13.7 | −14.7 ± 17.5 | −9.7 ± 15.4 | −17.9 ± 14.9 | −9.2 ± 11.5 | −10.2 ± 7.8 | −11.6 ± 11.7 |
| 95% CI | −16.8, −6.9 | −13.9, −5.9 | −19.5, −10.4 | −23.4, −6.0 | −16.9, −2.4 | −24.8, −10.9 | −14.7, −3.6 | −14.1, −6.4 | −17.6, −5.6 |
| *p*-value | <0.001 | <0.001 | <0.001 | 0.002 | 0.011 | <0.001 | 0.003 | <0.001 | <0.001 |
| EOT, *n* | 40 | 40 | 39 | 21 | 20 | 20 | 19 | 20 | 19 |
| Mean ± SD | 128.6 ± 11.3 | 129.5 ± 13.9 | 130.2 ± 12.2 | 127.9 ± 11.7 | 128.9 ± 15.1 | 126.4 ± 12.9 | 129.3 ± 11.1 | 130.2 ± 12.8 | 134.3 ± 10.1 |
| Change from baseline | −12.0 ± 14.5 | −9.7 ± 12.7 | −14.6 ± 13.5 | −14.5 ± 16.5 | −9.7 ± 15.4 | −17.9 ± 14.9 | −9.2 ± 11.5 | −9.8 ± 9.8 | −11.1 ± 11.2 |
| 95% CI | −16.6, −7.3 | −13.8, −5.6 | −18.9, −10.2 | −22.0, −6.9 | −16.9, −2.4 | −24.8, −10.9 | −14.7, −3.6 | −14.3, −5.2 | −16.5, −5.7 |
| *p*-value | <0.001 | <0.001 | <0.001 | <0.001 | 0.011 | <0.001 | 0.003 | <0.001 | <0.001 |
| Office DBP, mmHg |  |  |  |  |  |  |  |  |  |
| Baseline, *n* | 40 | 41 | 40 | 21 | 21 | 20 | 19 | 20 | 20 |
| Mean ± SD | 86.7 ± 11.8 | 83.0 ± 9.6 | 90.1 ± 10.4 | 85.5 ± 13.8 | 79.9 ± 8.7 | 87.9 ± 12.4 | 87.9 ± 9.3 | 86.4 ± 9.5 | 92.4 ± 7.8 |
| Week 12, *n* | 37 | 38 | 37 | 18 | 20 | 20 | 19 | 18 | 17 |
| Mean ± SD | 78.6 ± 9.9 | 78.1 ± 12.9 | 80.0 ± 11.2 | 75.8 ± 12.1 | 75.0 ± 10.0 | 76.5 ± 11.2 | 81.2 ± 6.7 | 81.6 ± 15.0 | 84.2 ± 9.9 |
| Change from baseline | −8.5 ± 7.3 | −5.1 ± 9.9 | −10.1 ± 8.8 | −10.3 ± 8.2 | −5.0 ± 10.0 | −11.4 ± 9.2 | −6.7 ± 6.0 | −5.2 ± 10.1 | −8.6 ± 8.2 |
| 95% CI | −10.9, −6.0 | −8.3, −1.8 | −13.1, −7.2 | −14.4, −6.3 | −9.6, −0.3 | −15.7, −7.1 | −9.6, −3.8 | −10.2, −0.1 | −12.9, −4.4 |
| *p*-value | <0.001 | 0.003 | <0.001 | <0.001 | 0.039 | <0.001 | <0.001 | 0.044 | <0.001 |
| EOT, *n* | 40 | 40 | 39 | 21 | 20 | 20 | 19 | 20 | 19 |
| Mean ± SD | 77.8 ± 10.3 | 78.2 ± 12.7 | 80.4 ± 11.0 | 74.7 ± 12.0 | 75.0 ± 10.0 | 76.5 ± 11.2 | 81.2 ± 6.7 | 81.4 ± 14.5 | 84.5 ± 9.4 |
| Change from baseline | −8.9 ± 7.2 | −5.0 ± 9.8 | −9.9 ± 8.6 | −10.9 ± 7.7 | −5.0 ± 10.0 | −11.4 ± 9.2 | −6.7 ± 6.0 | −5.0 ± 9.8 | −8.3 ± 7.9 |
| 95% CI | −11.2, −6.6 | −8.1, −1.8 | −12.7, −7.1 | −14.4, −7.3 | −9.6, −0.3 | −15.7, −7.1 | −9.6, −3.8 | −9.5, −0.4 | −12.0, −4.5 |
| *p*-value | <0.001 | 0.003 | <0.001 | <0.001 | 0.039 | <0.001 | <0.001 | 0.035 | <0.001 |

^a^ This was a *post hoc* analysis.

*ARB* angiotensin receptor blocker, *BP* blood pressure, *CCB* calcium channel blocker, *CI* confidence interval, *DBP* diastolic blood pressure, *EOT* end of treatment, *K* potassium, *Na* sodium, *SBP* systolic blood pressure.

**Table S11.** BP according to baseline estimated 24-h urinary Na excretion (per protocol set)^a^

|  | **Total**  ***N* = 121** | | | **ARB subcohort**  ***n* = 62** | | | **CCB subcohort**  ***n* = 59** | | |
| --- | --- | --- | --- | --- | --- | --- | --- | --- | --- |
| **Estimated 24-h urinary Na excretion** | **Lower tertile**^b^ | **Middle tertile**^c^ | **Higher tertile**^d^ | **Lower tertile**^b^ | **Middle tertile**^c^ | **Higher tertile**^d^ | **Lower tertile**^b^ | **Middle tertile**^c^ | **Higher tertile**^d^ |
| Morning home SBP, mmHg |  |  |  |  |  |  |  |  |  |
| Baseline, *n* | 40 | 41 | 40 | 22 | 22 | 18 | 18 | 19 | 22 |
| Mean ± SD | 134.9 ± 14.3 | 138.5 ± 11.5 | 136.6 ± 10.5 | 135.0 ± 13.1 | 140.1 ± 13.1 | 136.6 ± 11.6 | 134.7 ± 16.0 | 136.7 ± 9.3 | 136.6 ± 9.8 |
| Week 12, *n* | 38 | 39 | 35 | 20 | 20 | 18 | 18 | 19 | 17 |
| Mean ± SD | 122.8 ± 11.4 | 126.2 ± 9.4 | 125.9 ± 10.6 | 121.4 ± 9.1 | 124.9 ± 11.0 | 123.8 ± 9.4 | 124.5 ± 13.5 | 127.6 ± 7.4 | 128.2 ± 11.7 |
| Change from baseline | −12.0 ± 12.0 | −12.7 ± 11.7 | −11.5 ± 8.5 | −13.7 ± 13.6 | −16.1 ± 14.6 | −12.7 ± 8.8 | −10.2 ± 10.1 | −9.1 ± 6.2 | −10.2 ± 8.3 |
| 95% CI | −16.0, −8.1 | −16.5, −8.9 | −14.4, −8.6 | −20.0, −7.4 | −22.9, −9.2 | −17.1, −8.3 | −15.2, −5.2 | −12.1, −6.1 | −14.5, −6.0 |
| *p*-value | <0.001 | <0.001 | <0.001 | <0.001 | <0.001 | <0.001 | <0.001 | <0.001 | <0.001 |
| EOT, *n* | 40 | 40 | 39 | 22 | 21 | 18 | 18 | 19 | 21 |
| Mean ± SD | 123.2 ± 11.3 | 125.8 ± 9.6 | 126.1 ± 10.2 | 122.1 ± 9.4 | 124.2 ± 11.2 | 123.8 ± 9.4 | 124.5 ± 13.5 | 127.6 ± 7.4 | 128.1 ± 10.7 |
| Change from baseline | −11.7 ± 11.9 | −13.0 ± 11.7 | −10.5 ± 8.7 | −12.9 ± 13.3 | −16.5 ± 14.4 | −12.7 ± 8.8 | −10.2 ± 10.1 | −9.1 ± 6.2 | −8.7 ± 8.4 |
| 95% CI | −15.5, −7.8 | −16.7, −9.2 | −13.4, −7.7 | −18.8, −7.0 | −23.0, −9.9 | −17.1, −8.3 | −15.2, −5.2 | −12.1, −6.1 | −12.5, −4.9 |
| *p*-value | <0.001 | <0.001 | <0.001 | <0.001 | <0.001 | <0.001 | <0.001 | <0.001 | <0.001 |
| Morning home DBP, mmHg |  |  |  |  |  |  |  |  |  |
| Baseline, *n* | 40 | 41 | 40 | 22 | 22 | 18 | 18 | 19 | 22 |
| Mean ± SD | 86.7 ± 9.8 | 90.5 ± 9.4 | 87.1 ± 6.8 | 85.3 ± 8.4 | 92.1 ± 10.1 | 85.9 ± 6.2 | 88.3 ± 11.3 | 88.6 ± 8.3 | 88.0 ± 7.2 |
| Week 12, *n* | 38 | 39 | 35 | 20 | 20 | 18 | 18 | 19 | 17 |
| Mean ± SD | 80.8 ± 8.8 | 83.1 ± 8.0 | 81.1 ± 7.3 | 77.9 ± 8.5 | 82.1 ± 7.3 | 78.1 ± 5.7 | 84.1 ± 8.2 | 84.2 ± 8.7 | 84.3 ± 7.7 |
| Change from baseline | −6.0 ± 6.4 | −7.4 ± 8.5 | −6.5 ± 3.9 | −7.6 ± 6.5 | −10.2 ± 10.2 | −7.8 ± 3.4 | −4.2 ± 5.9 | −4.5 ± 4.8 | −5.1 ± 4.1 |
| 95% CI | −8.1, −3.9 | −10.2, −4.7 | −7.8, −5.1 | −10.6, −4.6 | −15.0, −5.4 | −9.5, −6.1 | −7.1, −1.3 | −6.8, −2.1 | −7.2, −3.0 |
| *p*-value | <0.001 | <0.001 | <0.001 | <0.001 | <0.001 | <0.001 | 0.007 | <0.001 | <0.001 |
| EOT, *n* | 40 | 40 | 39 | 22 | 21 | 18 | 18 | 19 | 21 |
| Mean ± SD | 81.1 ± 8.7 | 82.9 ± 7.9 | 81.3 ± 7.1 | 78.7 ± 8.5 | 81.8 ± 7.2 | 78.1 ± 5.7 | 84.1 ± 8.2 | 84.2 ± 8.7 | 84.1 ± 7.2 |
| Change from baseline | −5.6 ± 6.5 | −7.6 ± 8.4 | −5.9 ± 4.4 | −6.6 ± 6.9 | −10.4 ± 10.0 | −7.8 ± 3.4 | −4.2 ± 5.9 | −4.5 ± 4.8 | −4.3 ± 4.7 |
| 95% CI | −7.6, −3.5 | −10.3, −4.9 | −7.4, −4.5 | −9.7, −3.6 | −15.0, −5.9 | −9.5, −6.1 | −7.1, −1.3 | −6.8, −2.1 | −6.5, −2.2 |
| *p*-value | <0.001 | <0.001 | <0.001 | <0.001 | <0.001 | <0.001 | 0.007 | <0.001 | <0.001 |
| Bedtime home SBP, mmHg |  |  |  |  |  |  |  |  |  |
| Baseline, *n* | 40 | 41 | 40 | 22 | 22 | 18 | 18 | 19 | 22 |
| Mean ± SD | 131.8 ± 14.0 | 131.4 ± 13.9 | 129.9 ± 12.5 | 131.3 ± 15.1 | 133.1 ± 17.0 | 130.6 ± 12.7 | 132.3 ± 13.0 | 129.5 ± 9.2 | 129.3 ± 12.7 |
| Week 12, *n* | 38 | 39 | 35 | 20 | 20 | 18 | 18 | 19 | 17 |
| Mean ± SD | 120.4 ± 11.0 | 121.0 ± 12.6 | 120.6 ± 10.5 | 117.3 ± 10.3 | 118.3 ± 14.3 | 117.9 ± 9.8 | 123.8 ± 11.0 | 123.8 ± 10.1 | 123.4 ± 10.7 |
| Change from baseline | −11.3 ± 11.2 | −11.0 ± 11.8 | −9.7 ± 7.6 | −13.7 ± 13.1 | −16.1 ± 13.1 | −12.6 ± 7.0 | −8.6 ± 8.2 | −5.7 ± 7.5 | −6.6 ± 7.1 |
| 95% CI | −15.0, −7.6 | −14.9, −7.2 | −12.3, −7.1 | −19.8, −7.6 | −22.2, −10.0 | −16.1, −9.1 | −12.6, −4.5 | −9.3, −2.1 | −10.2, −2.9 |
| *p*-value | <0.001 | <0.001 | <0.001 | <0.001 | <0.001 | <0.001 | <0.001 | 0.004 | 0.002 |
| EOT, *n* | 40 | 40 | 39 | 22 | 21 | 18 | 18 | 19 | 21 |
| Mean ± SD | 120.3 ± 10.9 | 120.5 ± 12.9 | 120.8 ± 9.9 | 117.5 ± 10.2 | 117.4 ± 14.5 | 117.9 ± 9.8 | 123.8 ± 11.0 | 123.8 ± 10.1 | 123.2 ± 9.7 |
| Change from baseline | −11.5 ± 11.2 | −11.5 ± 12.0 | −9.1 ± 7.6 | −13.8 ± 12.8 | −16.7 ± 13.1 | −12.6 ± 7.0 | −8.6 ± 8.2 | −5.7 ± 7.5 | −6.1 ± 6.9 |
| 95% CI | −15.0, −7.9 | −15.3, −7.6 | −11.6, −6.7 | −19.5, −8.1 | −22.7, −10.8 | −16.1, −9.1 | −12.6, −4.5 | −9.3, −2.1 | −9.3, −3.0 |
| *p*-value | <0.001 | <0.001 | <0.001 | <0.001 | <0.001 | <0.001 | <0.001 | 0.004 | <0.001 |
| Bedtime home DBP, mmHg |  |  |  |  |  |  |  |  |  |
| Baseline, *n* | 40 | 41 | 40 | 22 | 22 | 18 | 18 | 19 | 22 |
| Mean ± SD | 82.8 ± 9.0 | 84.0 ± 9.0 | 80.2 ± 7.7 | 81.3 ± 8.5 | 84.5 ± 9.6 | 79.6 ± 7.8 | 84.5 ± 9.6 | 83.3 ± 8.5 | 80.6 ± 7.7 |
| Week 12, *n* | 38 | 39 | 35 | 20 | 20 | 18 | 18 | 19 | 17 |
| Mean ± SD | 76.3 ± 9.8 | 77.2 ± 8.5 | 75.9 ± 8.1 | 72.9 ± 9.5 | 75.0 ± 7.9 | 72.6 ± 8.3 | 80.1 ± 9.0 | 79.5 ± 8.6 | 79.5 ± 6.3 |
| Change from baseline | −6.4 ± 7.3 | −6.9 ± 7.7 | −4.6 ± 4.4 | −8.3 ± 8.0 | −9.9 ± 8.2 | −7.0 ± 3.3 | −4.4 ± 6.0 | −3.8 ± 5.8 | −2.0 ± 4.0 |
| 95% CI | −8.8, −4.1 | −9.4, −4.4 | −6.1, −3.1 | −12.0, −4.6 | −13.7, −6.0 | −8.7, −5.3 | −7.4, −1.4 | −6.6, −1.1 | −4.1, 0.1 |
| *p*-value | <0.001 | <0.001 | <0.001 | <0.001 | <0.001 | <0.001 | 0.006 | 0.010 | 0.056 |
| EOT, *n* | 40 | 40 | 39 | 22 | 21 | 18 | 18 | 19 | 21 |
| Mean ± SD | 76.6 ± 9.7 | 77.0 ± 8.4 | 76.3 ± 7.8 | 73.7 ± 9.4 | 74.8 ± 7.8 | 72.6 ± 8.3 | 80.1 ± 9.0 | 79.5 ± 8.6 | 79.5 ± 5.7 |
| Change from baseline | −6.2 ± 7.2 | −7.2 ± 7.7 | −3.9 ± 5.0 | −7.6 ± 7.9 | −10.1 ± 8.1 | −7.0 ± 3.3 | −4.4 ± 6.0 | −3.8 ± 5.8 | −1.2 ± 4.7 |
| 95% CI | −8.5, −3.8 | −9.6, −4.7 | −5.5, −2.3 | −11.1, −4.1 | −13.8, −6.4 | −8.7, −5.3 | −7.4, −1.4 | −6.6, −1.1 | −3.4, 0.9 |
| *p*-value | <0.001 | <0.001 | <0.001 | <0.001 | <0.001 | <0.001 | 0.006 | 0.010 | 0.237 |
| Office SBP, mmHg |  |  |  |  |  |  |  |  |  |
| Baseline, *n* | 40 | 41 | 40 | 22 | 22 | 18 | 18 | 19 | 22 |
| Mean ± SD | 137.9 ± 13.4 | 139.7 ± 13.1 | 147.4 ± 17.7 | 136.0 ± 13.8 | 140.4 ± 15.4 | 149.4 ± 20.8 | 140.2 ± 12.9 | 138.8 ± 10.2 | 145.7 ± 15.0 |
| Week 12, *n* | 38 | 39 | 35 | 20 | 20 | 18 | 18 | 19 | 17 |
| Mean ± SD | 127.2 ± 11.8 | 127.8 ± 11.1 | 133.5 ± 13.2 | 125.7 ± 12.3 | 126.1 ± 12.5 | 133.1 ± 14.6 | 128.9 ± 11.3 | 129.6 ± 9.4 | 133.9 ± 12.0 |
| Change from baseline | −10.8 ± 15.0 | −12.5 ± 11.7 | −13.5 ± 14.3 | −10.4 ± 18.3 | −15.6 ± 12.6 | −16.4 ± 16.8 | −11.3 ± 10.9 | −9.2 ± 9.9 | −10.5 ± 10.7 |
| 95% CI | −15.7, −5.8 | −16.2, −8.7 | −18.4, −8.6 | −18.9, −1.8 | −21.5, −9.7 | −24.8, −8.0 | −16.7, −5.8 | −13.9, −4.4 | −16.0, −5.0 |
| *p*-value | <0.001 | <0.001 | <0.001 | 0.020 | <0.001 | <0.001 | <0.001 | <0.001 | 0.001 |
| EOT, *n* | 40 | 40 | 39 | 22 | 21 | 18 | 18 | 19 | 21 |
| Mean ± SD | 126.8 ± 11.7 | 127.7 ± 11.0 | 133.9 ± 13.5 | 125.0 ± 11.9 | 125.9 ± 12.2 | 133.1 ± 14.6 | 128.9 ± 11.3 | 129.6 ± 9.4 | 134.7 ± 12.8 |
| Change from baseline | −11.1 ± 14.9 | −12.3 ± 11.5 | −12.8 ± 14.4 | −10.9 ± 17.8 | −15.2 ± 12.4 | −16.4 ± 16.8 | −11.3 ± 10.9 | −9.2 ± 9.9 | −9.7 ± 11.5 |
| 95% CI | −15.8, −6.3 | −16.0, −8.6 | −17.4, −8.1 | −18.8, −3.0 | −20.8, −9.5 | −24.8, −8.0 | −16.7, −5.8 | −13.9, −4.4 | −14.9, −4.4 |
| *p*-value | <0.001 | <0.001 | <0.001 | 0.009 | <0.001 | <0.001 | <0.001 | <0.001 | 0.001 |
| Office DBP, mmHg |  |  |  |  |  |  |  |  |  |
| Baseline, *n* | 40 | 41 | 40 | 22 | 22 | 18 | 18 | 19 | 22 |
| Mean ± SD | 84.7 ± 11.6 | 86.4 ± 10.5 | 88.6 ± 10.5 | 81.1 ± 12.4 | 85.5 ± 11.5 | 87.0 ± 12.2 | 89.0 ± 9.1 | 87.5 ± 9.5 | 90.0 ± 9.0 |
| Week 12, *n* | 38 | 39 | 35 | 20 | 20 | 18 | 18 | 19 | 17 |
| Mean ± SD | 77.2 ± 9.3 | 79.1 ± 13.1 | 80.6 ± 11.3 | 73.2 ± 9.9 | 75.2 ± 11.4 | 79.2 ± 11.2 | 81.6 ± 6.2 | 83.2 ± 13.9 | 82.1 ± 11.6 |
| Change from baseline | −7.6 ± 8.5 | −7.7 ± 9.7 | −8.3 ± 8.7 | −7.8 ± 10.7 | −10.9 ± 7.7 | −7.8 ± 10.0 | −7.4 ± 5.3 | −4.4 ± 10.6 | −8.8 ± 7.4 |
| 95% CI | −10.4, −4.8 | −10.8, −4.6 | −11.3, −5.3 | −12.8, −2.7 | −14.5, −7.2 | −12.8, −2.9 | −10.1, −4.8 | −9.5, 0.7 | −12.6, −5.0 |
| *p*-value | <0.001 | <0.001 | <0.001 | 0.004 | <0.001 | 0.004 | <0.001 | 0.089 | <0.001 |
| EOT, *n* | 40 | 40 | 39 | 22 | 21 | 18 | 18 | 19 | 21 |
| Mean ± SD | 76.8 ± 9.4 | 78.7 ± 13.2 | 80.8 ± 11.0 | 73.0 ± 10.0 | 74.6 ± 11.4 | 79.2 ± 11.2 | 81.6 ± 6.2 | 83.2 ± 13.9 | 82.3 ± 10.8 |
| Change from baseline | −7.8 ± 8.3 | −8.0 ± 9.7 | −7.9 ± 8.5 | −8.1 ± 10.2 | −11.2 ± 7.7 | −7.8 ± 10.0 | −7.4 ± 5.3 | −4.4 ± 10.6 | −7.9 ± 7.2 |
| 95% CI | −10.5, −5.2 | −11.0, −4.9 | −10.6, −5.1 | −12.7, −3.6 | −14.7, −7.7 | −12.8, −2.9 | −10.1, −4.8 | −9.5, 0.7 | −11.2, −4.6 |
| *p*-value | <0.001 | <0.001 | <0.001 | 0.001 | <0.001 | 0.004 | <0.001 | 0.089 | <0.001 |

^a^ This was a *post hoc* analysis.

^b^ Lower tertile, <142.4 mEq/day.

^c^ Middle tertile, 142.4 to 190.1 mEq/day.

^d^ Higher tertile, ≥190.1 mEq/day.

*ARB* angiotensin receptor blocker, *BP* blood pressure, *CCB* calcium channel blocker, *CI* confidence interval, *DBP* diastolic blood pressure, *EOT* end of treatment, *Na* sodium, *SBP* systolic blood pressure.

**Table S12.** Change in UACR and NT-proBNP from baseline to Week 12 in the total population and ARB and CCB subcohorts (full analysis set)

| **Variables** | **Total**  ***N* = 126** | **ARB** **subcohort**  ***n* = 67** | **CCB** **subcohort**  ***n* = 59** |
| --- | --- | --- | --- |
| UACR, mg/gCr |  |  |  |
| Baseline, *n* | 124 | 66 | 58 |
| Mean ± SD | 34.6 ± 130.8 | 44.1 ± 170.8 | 23.9 ± 58.5 |
| Week 12, *n* | 107 | 59 | 48 |
| Mean ± SD | 17.0 ± 48.2 | 17.0 ± 55.6 | 17.0 ± 37.9 |
| Change from baseline | −21.0 ± 96.2 | −30.1 ± 126.9 | −9.9 ± 27.9 |
| Geometric percentage change from baseline  95% CI | −37.0  [−46.1, −26.4]*** | −42.0  [−54.1, −26.8]*** | −30.3  [−43.0, −14.8]*** |
| NT-proBNP, pg/mL |  |  |  |
| Baseline, *n* | 117 | 60 | 57 |
| Mean ± SD | 59.9 ± 61.5 | 72.6 ± 79.6 | 46.5 ± 28.3 |
| Week 12, *n* | 95 | 50 | 45 |
| Mean ± SD | 52.6 ± 53.7 | 63.2 ± 68.6 | 40.8 ± 25.8 |
| Change from baseline | −12.1 ± 27.5 | −14.3 ± 33.9 | −9.7 ± 18.0 |
| Geometric percentage change from baseline  95% CI | −21.5  [−28.5, −13.8]*** | −21.3  [−32.1, −8.9]** | −21.7  [−30.3, −12.0]*** |

***p* <0.01, ****p* <0.001 vs baseline, paired *t*-test.

*p-*values are only presented for geometric percentage change from baseline.

*ARB* angiotensin receptor blocker, *CCB* calcium channel blocker, *CI* confidence interval, *NT-proBNP* N-terminal pro-brain natriuretic peptide, *UACR* urinary albumin-to-creatinine ratio.

## Table S13. Change in UACR and NT-proBNP from baseline to Week 12 in the total population and ARB and CCB subcohorts (per protocol set)

| **Variables** | **Total**  ***N* = 121** | **ARB subcohort**  ***n* = 62** | **CCB subcohort**  ***n* = 59** |
| --- | --- | --- | --- |
| UACR, mg/gCr |  |  |  |
| Baseline, *n* | 119 | 61 | 58 |
| Mean ± SD | 35.0 ± 133.5 | 45.6 ± 177.7 | 23.9 ± 58.5 |
| Week 12, *n* | 103 | 55 | 48 |
| Mean ± SD | 16.9 ± 49.1 | 16.8 ± 57.5 | 17.0 ± 37.9 |
| Change from baseline | −21.6 ± 98.0 | −31.8 ± 131.3 | −9.9 ± 27.9 |
| Geometric percentage change from baseline  95% CI | −37.4  [−46.7, −26.5]*** | −43.0  [−55.6, −26.9]*** | −30.3  [−43.0, −14.8]*** |
| NT-proBNP, pg/mL |  |  |  |
| Baseline, *n* | 114 | 57 | 57 |
| Mean ± SD | 59.8 ± 62.0 | 73.1 ± 81.2 | 46.5 ± 28.3 |
| Week 12, *n* | 93 | 48 | 45 |
| Mean ± SD | 53.3 ± 54.0 | 65.1 ± 69.2 | 40.8 ± 25.8 |
| Change from baseline | −12.0 ± 27.8 | −14.1 ± 34.6 | −9.7 ± 18.0 |
| Geometric percentage change from baseline  95% CI | −20.4  [−27.5, −12.7]*** | −19.3  [−30.4, −6.4]** | −21.7  [−30.3, −12.0]*** |

***p* <0.01, ****p* <0.001 vs baseline, paired *t*-test.

*p-*values are only presented for geometric percentage change from baseline.

*ARB* angiotensin receptor blocker, *CCB* calcium channel blocker, *CI* confidence interval, *NT-proBNP* N-terminal pro-brain natriuretic peptide, *UACR* urinary albumin-to-creatinine ratio.

**Table S14.** Change in biomarker data from baseline to Week 12 in the total population and ARB and CCB subcohorts (full analysis set)

| **Variables** |  | **Total**  ***N* = 126** | **ARB subcohort**  ***n* = 67** | **CCB subcohort**  ***n* = 59** |
| --- | --- | --- | --- | --- |
| PAC, pg/mL |  |  |  |  |
| Baseline | Mean ± SD | 39.4 ± 26.8 | 36.2 ± 21.3 | 42.1 ± 30.7 |
| Week 12 | Mean ± SD | 69.7 ± 55.9 | 52.6 ± 42.8 | 88.0 ± 62.6 |
|  | Change from baseline | 41.6 ± 44.0 | 30.3 ± 39.8 | 51.0 ± 45.4 |
| PRA, ng/mL/h |  |  |  |  |
| Baseline | Mean ± SD | 4.2 ± 12.8 | 6.7 ± 17.0 | 1.3 ± 1.3 |
| Week 12 | Mean ± SD | 7.3 ± 13.4 | 11.7 ± 17.3 | 2.4 ± 2.0 |
|  | Change from baseline | 3.1 ± 14.5 | 4.8 ± 19.7 | 1.2 ± 1.4 |
| Urinary Na, mEq/L |  |  |  |  |
| Baseline | Mean ± SD | 108.0 ± 52.7 | 102.6 ± 50.1 | 114.2 ± 55.2 |
| Week 12 | Mean ± SD | 124.0 ± 64.9 | 119.2 ± 61.5 | 129.6 ± 68.7 |
|  | Change from baseline | 14.6 ± 62.0 | 14.0 ± 55.3 | 15.4 ± 69.4 |
| Urinary K, mEq/L |  |  |  |  |
| Baseline | Mean ± SD | 42.5 ± 23.5 | 41.3 ± 23.9 | 43.8 ± 23.0 |
| Week 12 | Mean ± SD | 43.5 ± 23.6 | 41.4 ± 23.5 | 45.8 ± 23.8 |
|  | Change from baseline | 0.9 ± 24.2 | 0.3 ± 22.3 | 1.5 ± 26.3 |
| Urinary Na/K ratio |  |  |  |  |
| Baseline | Mean ± SD | 3.1 ± 1.9 | 3.1 ± 2.0 | 3.1 ± 1.7 |
| Week 12 | Mean ± SD | 3.5 ± 2.5 | 3.4 ± 1.8 | 3.5 ± 3.2 |
|  | Change from baseline | 0.3 ± 2.5 | 0.2 ± 1.6 | 0.5 ± 3.2 |

*P*-values were not calculated for changes from baseline to Week 12.

*ARB* angiotensin receptor blocker, *CCB* calcium channel blocker, *K* potassium, *Na* sodium, *PAC* plasma aldosterone concentration, *PRA* plasma renin activity.

**Table S15.** Change in biomarker data from baseline to Week 12 in the total population and ARB and CCB subcohorts (per protocol set)

| **Variables** |  | **Total**  ***N* = 121** | **ARB subcohort**  ***n* = 62** | **CCB subcohort**  ***n* = 59** |
| --- | --- | --- | --- | --- |
| PAC, pg/mL |  |  |  |  |
| Baseline | Mean ± SD | 39.2 ± 26.7 | 35.7 ± 20.6 | 42.1 ± 30.7 |
| Week 12 | Mean ± SD | 69.4 ± 55.8 | 51.1 ± 41.1 | 88.0 ± 62.6 |
|  | Change from baseline | 41.2 ± 42.2 | 28.8 ± 34.6 | 51.0 ± 45.4 |
| PRA, ng/mL/h |  |  |  |  |
| Baseline | Mean ± SD | 3.9 ± 12.7 | 6.3 ± 17.4 | 1.3 ± 1.3 |
| Week 12 | Mean ± SD | 6.4 ± 10.6 | 10.4 ± 13.7 | 2.4 ± 2.0 |
|  | Change from baseline | 2.6 ± 13.4 | 4.0 ± 18.5 | 1.2 ± 1.4 |
| Urinary Na, mEq/L |  |  |  |  |
| Baseline | Mean ± SD | 109.1 ± 52.5 | 104.2 ± 50.0 | 114.2 ± 55.2 |
| Week 12 | Mean ± SD | 125.4 ± 65.4 | 121.5 ± 62.5 | 129.6 ± 68.7 |
|  | Change from baseline | 15.4 ± 62.7 | 15.4 ± 56.3 | 15.4 ± 69.4 |
| Urinary K, mEq/L |  |  |  |  |
| Baseline | Mean ± SD | 43.3 ± 23.5 | 42.8 ± 24.1 | 43.8 ± 23.0 |
| Week 12 | Mean ± SD | 44.0 ± 23.8 | 42.4 ± 23.8 | 45.8 ± 23.8 |
|  | Change from baseline | 0.8 ± 24.3 | 0.1 ± 22.5 | 1.5 ± 26.3 |
| Urinary Na/K ratio |  |  |  |  |
| Baseline | Mean ± SD | 3.1 ± 1.9 | 3.1 ± 2.1 | 3.1 ± 1.7 |
| Week 12 | Mean ± SD | 3.5 ± 2.6 | 3.4 ± 1.8 | 3.5 ± 3.2 |
|  | Change from baseline | 0.3 ± 2.5 | 0.2 ± 1.6 | 0.5 ± 3.2 |

*P*-values were not calculated for changes from baseline to Week 12.

*ARB* angiotensin receptor blocker, *CCB* calcium channel blocker, *K* potassium, *Na* sodium, *PAC* plasma aldosterone concentration, *PRA* plasma renin activity.

**Table S16.** Incidence of serum potassium level ≥5.5 and ≥6.0 mEq/L (safety analysis set)

| **Serum K level** | **Total**  ***N* = 126** | **ARB subcohort**  ***n* = 67** | **CCB subcohort**  ***n* = 59** |
| --- | --- | --- | --- |
| Serum K ≥5.5 mEq/L | 9 (7.1) | 4 (6.0) | 5 (8.5) |
| Serum K ≥6.0 mEq/L | 2 (1.6) | 0 (0.0) | 2 (3.4) |

Data are *n* (%).

*ARB* angiotensin receptor blocker, *CCB* calcium channel blocker, *K* potassium.
