# Supplementary material for: Exploratory study on the relationship between urinary sodium/potassium ratio, salt intake, and the antihypertensive effect of esaxerenone: the ENaK Study
Source: Hypertens Res. 2024 Jan 11;47(4):835–48. doi: 10.1038/s41440-023-01519-0 (PMC10994843; doi:10.1038/s41440-023-01519-0)
Supplement: Supplementary file 3 — Supplementary Figures [file 41440_2023_1519_MOESM3_ESM.docx]

# Supplementary Materials

# Supplementary Figures

**
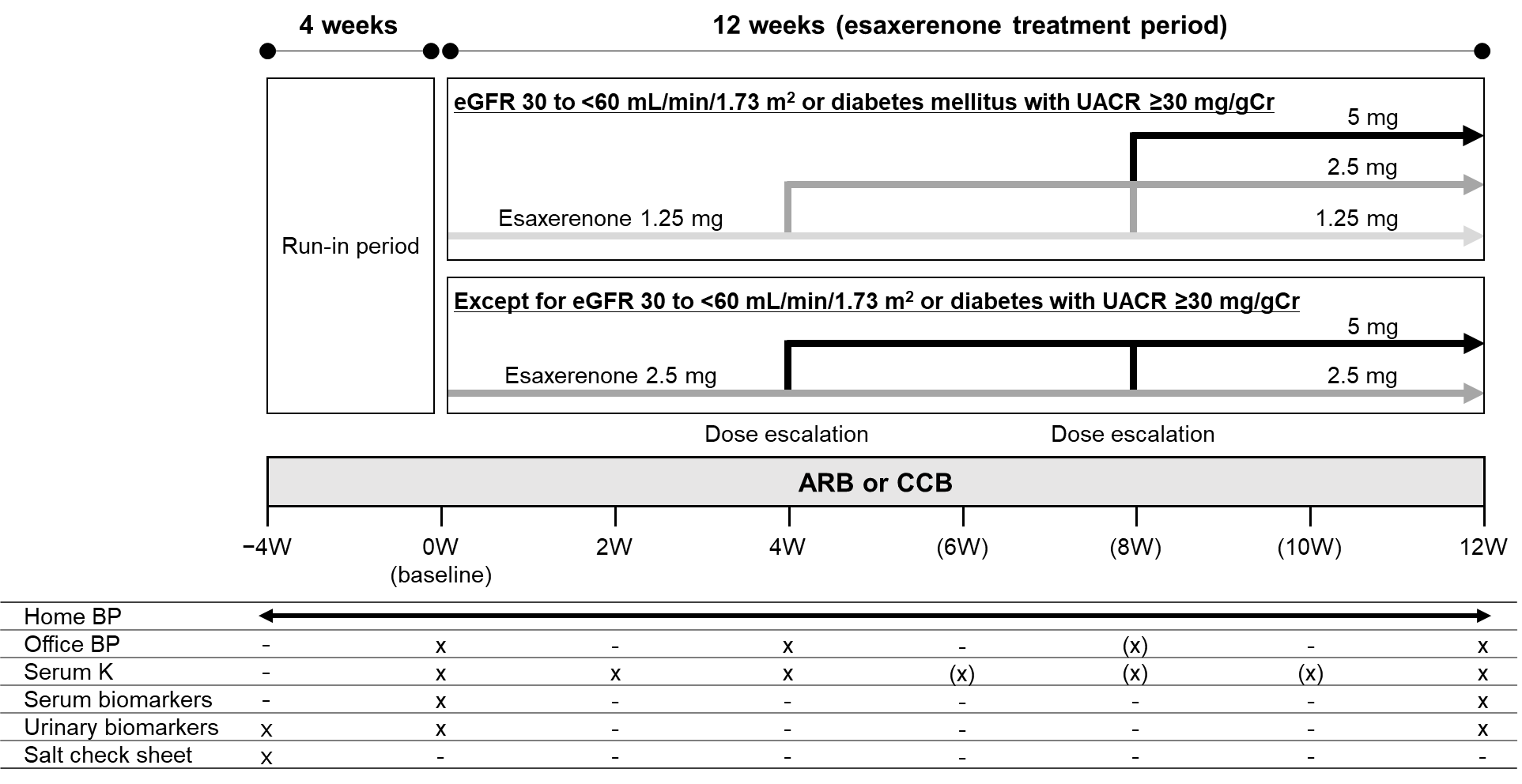
**

**Figure S1.** Study design

*ARB* angiotensin receptor blocker, *BP* blood pressure, *CCB* calcium channel blocker, *eGFR* creatinine-based estimated glomerular filtration rate*, K* potassium, *UACR* urinary albumin-to-creatinine ratio.

**
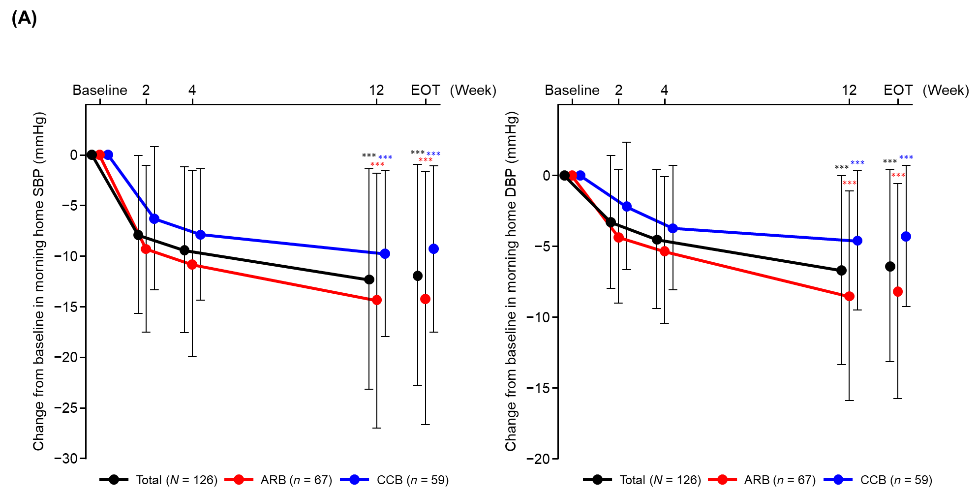
**

**
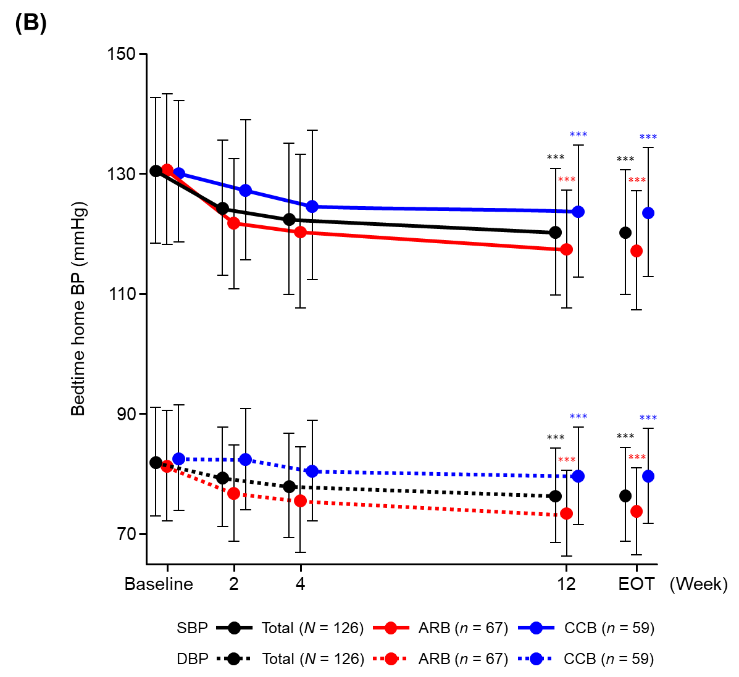
**

**
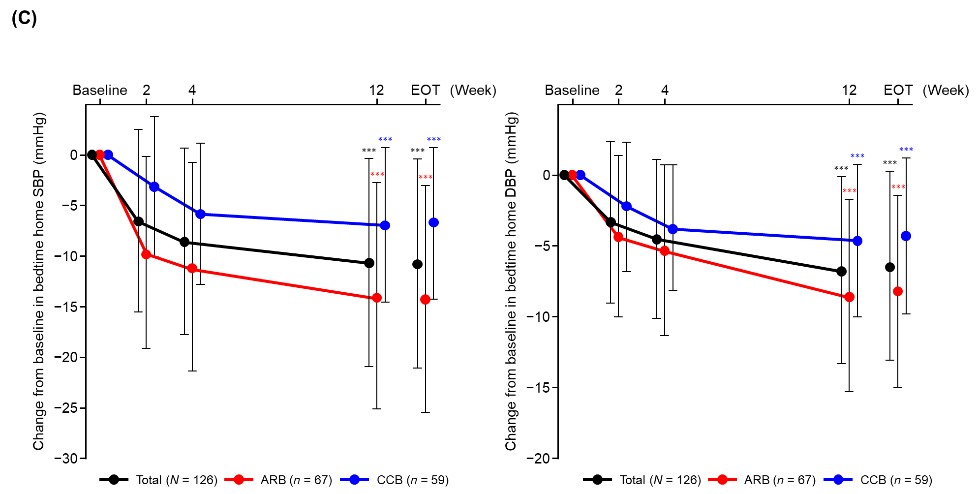
**

**
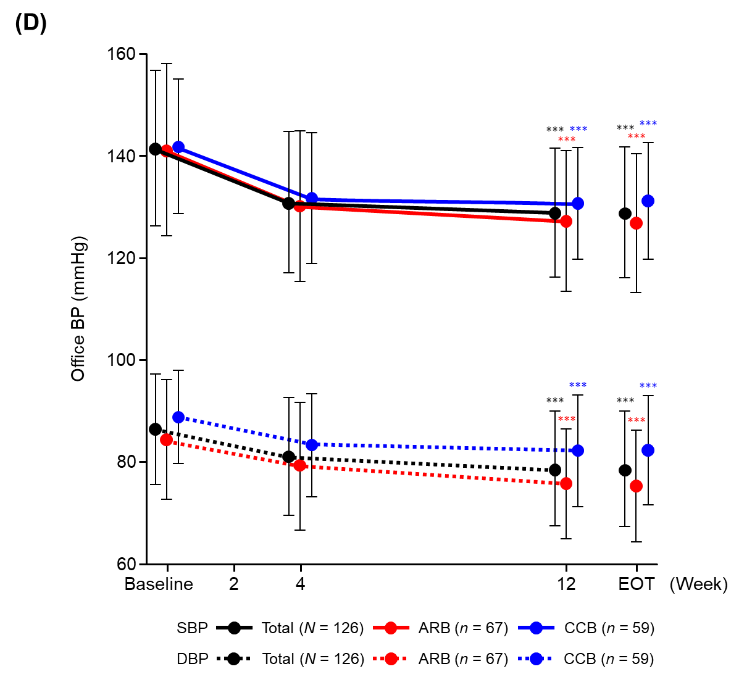
**

**
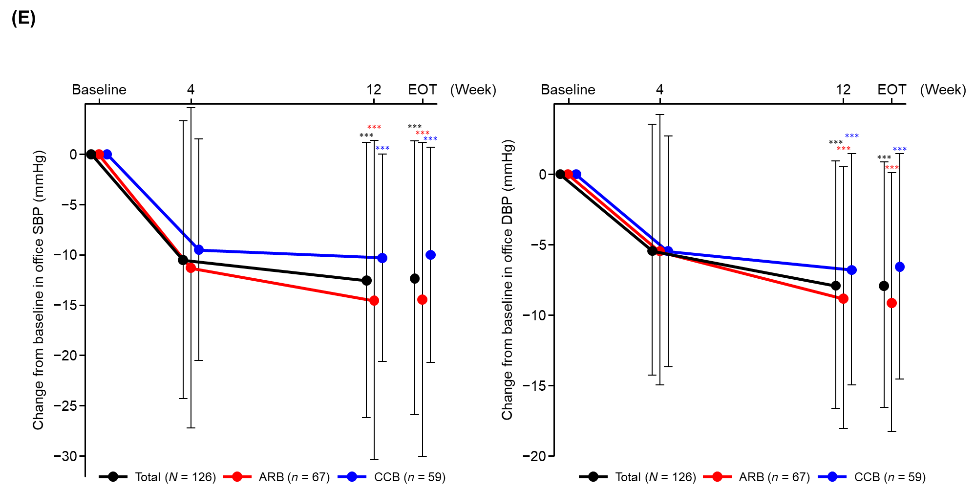
**

**Figure S2.** Time course change and changes from baseline in morning home BP (**A**), bedtime home BP (**B, C**), and office BP (**D, E**) (full analysis set).

Changes from baseline are shown in panels A, C, and E.

Time course changes are shown in panels B and D.

Data are mean ± SD.

****p* <0.001 vs baseline, paired *t*-test.

*ARB* angiotensin receptor blocker, *BP* blood pressure, *CCB* calcium channel blocker, *DBP* diastolic blood pressure, *EOT* end of treatment, *SBP* systolic blood pressure.


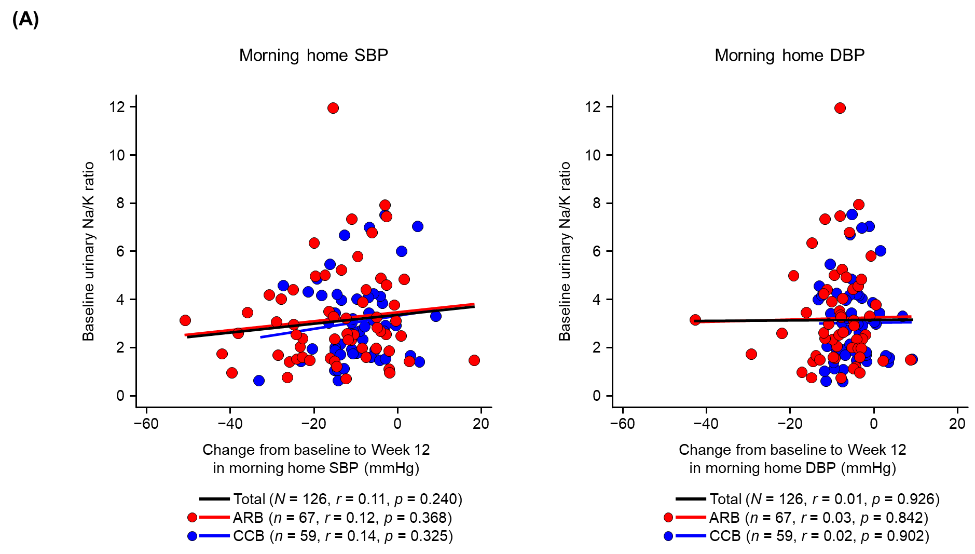


**
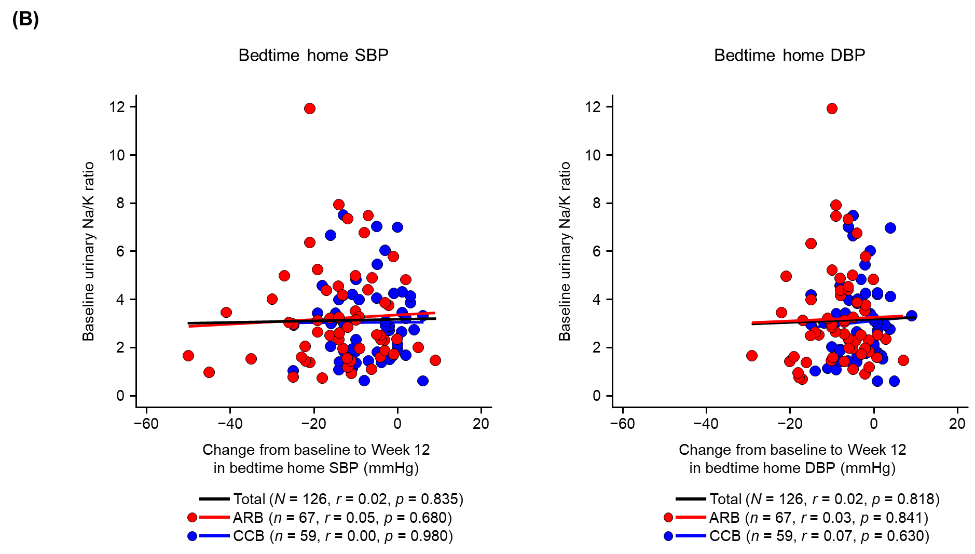
**

**
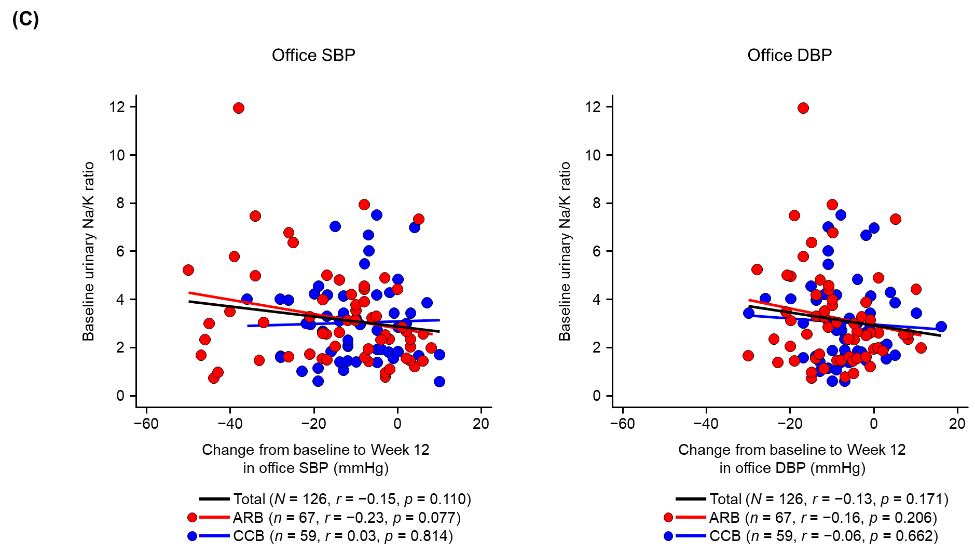
**

**
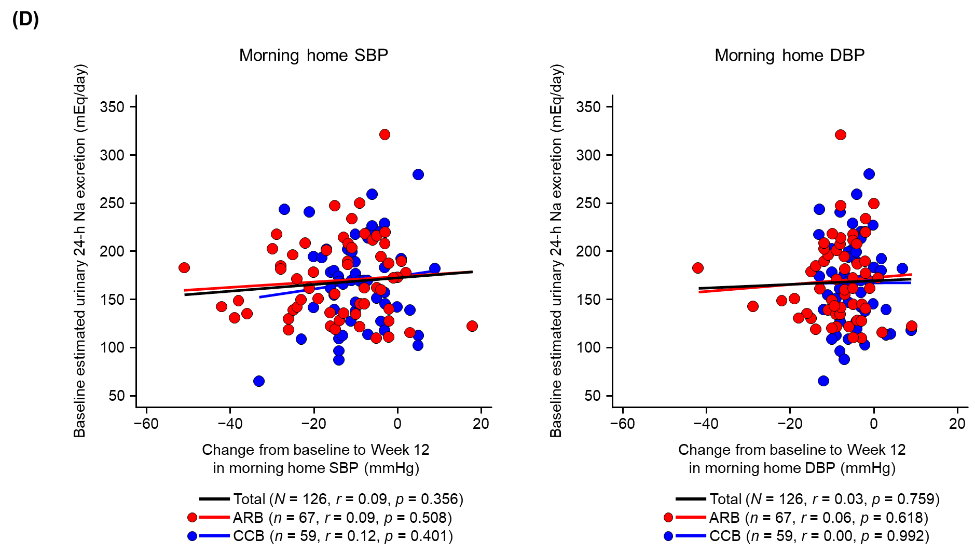
**

**
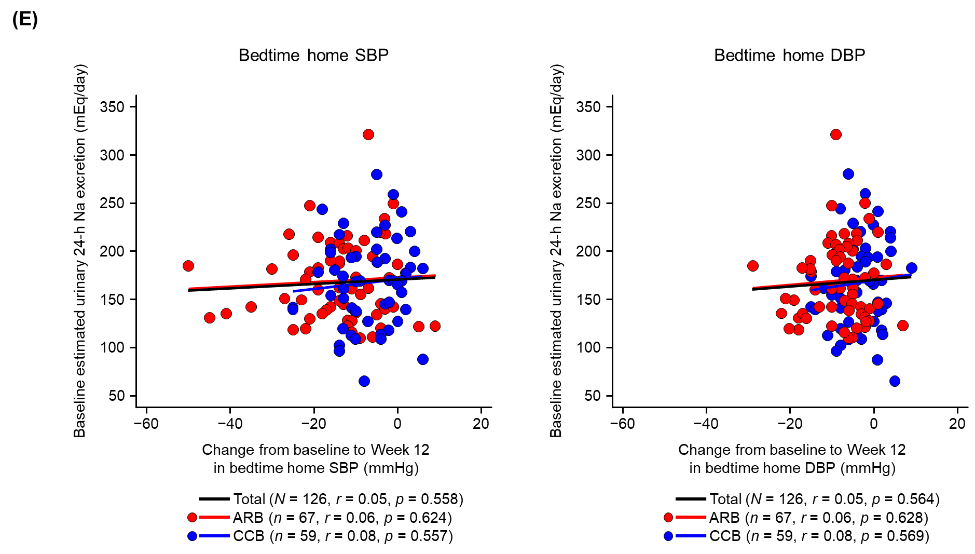
**

**
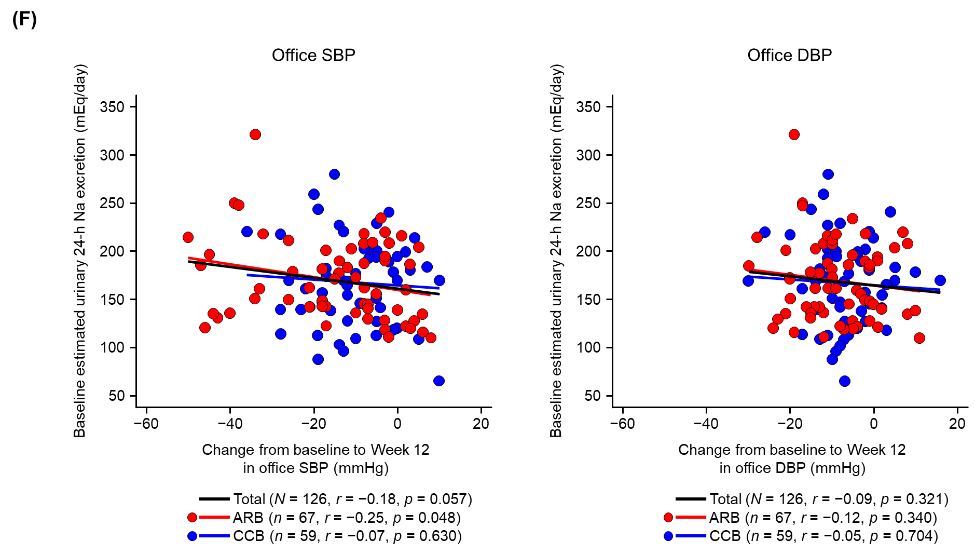
**

**Figure S3.** Correlation of change in BP from baseline to Week 12 with baseline urinary Na/K ratio (**A, B, C**: pre-specified analysis) and estimated 24-h urinary Na excretion (**D, E, F**: *post hoc* analysis) (full analysis set).

A, D: morning home BP. B, E: bedtime home BP. C, F: office BP.

*r*, Pearson’s correlation coefficient.

*ARB* angiotensin receptor blocker, *BP* blood pressure, *CCB* calcium channel blocker, *DBP* diastolic blood pressure, *K* potassium, *Na* sodium, *SBP* systolic blood pressure.

**
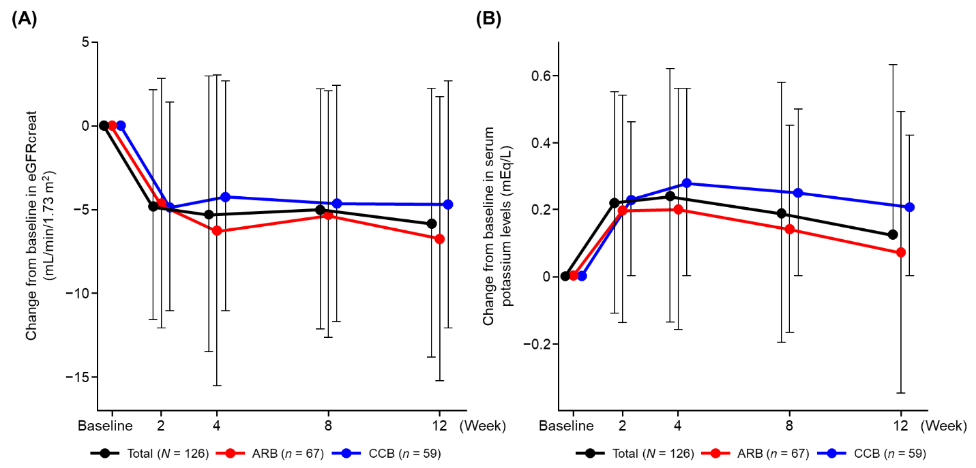
Figure S4.** Change from baseline in eGFR (**A**) and serum K levels (**B**) during the study period in the total population and ARB and CCB subcohorts (safety analysis set).

Data are mean ± SD.

*ARB* angiotensin receptor blocker, *CCB* calcium channel blocker, *eGFR* creatinine-based estimated glomerular filtration rate, *K* potassium.
